# Supplementary material for: The cost-effectiveness of scaling-up rapid point-of-care testing for early infant diagnosis of HIV in southern Zambia
Source: PLoS One. 2021 Mar 9;16(3):e0248217. doi: 10.1371/journal.pone.0248217 (PMC7943017; doi:10.1371/journal.pone.0248217)
Supplement: S1 Methods — This document describes in detail the methods used for the study and assumptions made in the analysis. (DOCX) [file pone.0248217.s002.docx]

**S1 Methods.**

**CONTENT:**

1. Algorithms
2. Implementation models
3. Model parameters
4. Early Infant Diagnosis (EID) and Novel Screening for Exposed Babies (NSEBA) studies
5. Cost calculation
6. Sensitivity analysis
7. **Testing algorithms**

We compared four point-of-care (PoC) testing algorithms using either the GeneXpert or the m-PIMA platform against the standard-of-care (SoC) algorithm for early infant diagnosis (EID) prior to antiretroviral treatment (ART) initiation. In every algorithm, testing is repeated up to three times – at birth, at 6-weeks old and at 6-months old – or until a positive test result. If the infant is not tested at birth, they may enter the cohort at 6-weeks or 6 months of age. Between testing time points, a proportion of infants are lost to follow-up, a proportion of HIV-uninfected infants become infected, and a proportion of HIV-infected infants experience HIV-related mortality until eventual ART initiation or end of follow-up (12 months of age). Once an infant enters the testing population they are assumed to receive recommended HIV care, including receipt of antiretroviral drugs for prophylaxis.

*Figure 1: SoC algorithm, Standard-of-care used for the initial and confirmatory test*

Enter testing population

**Testing**

**(SoC)**

Positive result^1^

Negative result^1^

ART starts within

60 days

ART doesn’t start within 60 days

Risk of transmission and mortality

Lost before next test

Return for next test

Remain on ART (delayed)

Positive result

*Lost before*

*results are*

*returned*

*Results*

*returned*

*Quickly*

*Results*

*not returned*

*quickly*

Remain on ART (rapid)

**Awaiting Results**

**of Second Test**

Never starts ART

ART starts after 60 days

Positive result

**Confirmatory testing**

**(SoC)**

*^1^ SoC is assumed to be 100% sensitive & specific*

Risk of mortality

Under the SoC, blood samples (dried blood spot cards, DBS) are collected in urban and rural healthcare centers (UHC and RHC) and transported to a central lab for testing. Due to delays in sample transportation, processing, and caregiver notification, not all caregivers receive timely results. When a positive result is received, a second blood sample is collected for confirmatory testing at the central lab and the infant initiates ART immediately.

For the purposes of the analysis and comparison, the sensitivity and specificity of PCR testing at the central lab was assumed to be 100%.

*Figure 2: PoC3 algorithm, Point-of-care test for the initial, confirmatory, and tie-breaker tests*

Never starts ART

Enter testing population

**Testing**

**(PoC)**

Positive result

Negative result

Positive result

Negative result

Risk of transmission or mortality^2^

Lost before next test

Return for next test

ART starts after 60 days

*Immediate 2^nd^ test*

*Lost before third test result*

ART starts within 60 days

Re-test

**Confirmatory testing**

**(PoC)**

*^2^ PoC specificity <100%; transmission possible among false positives*

**Tie-breaker testing**

**(PoC)**

**1-week delay**

Positive result

Negative result

ART does not start within 60 days

Risk of transmission or mortality^2^

ART starts after 60 days

ART does not start within 60 days

ART starts within 60 days

Blood samples are collected at the UHCs and RHCs and tested onsite using the PoC platform. Results are available and provided to the caregiver on the same day. When a positive result is received, a second sample is collected and tested onsite using the PoC platform (ART counseling may commence after the initial positive result). If the confirmatory PoC test is positive, the infant initiates ART immediately. If the confirmatory PoC test is negative, the caregiver is asked to return to the facility in a week with the infant to perform a tie-breaker PoC test. A one week interval was chosen as it is unlikely that three PoC tests could be performed on the same child in a given day and the infant may reside far from the clinic and be unable to return the next day. A small proportion of infants are lost to follow-up in this interval between the confirmatory and tie-breaker test. If the tie-breaker test is positive, ART is initiated immediately.

*Figure 3: PoC2+SoC algorithm, Point-of-care test as initial and confirmatory tests, standard-of-care as the tie-breaker test*

Never starts ART

Enter testing population

**Testing**

**(PoC)**

Positive result

Negative result

Positive result

Negative result

Risk of transmission or mortality^2^

Lost before next test

Return for next test

ART starts after 60 days

*Immediate 2^nd^ test*

*Lost before third test result*

ART starts within 60 days

Re-test

**Confirmatory testing**

**(PoC)**

*^2^ PoC specificity <100%; transmission possible among false positives*

**Tie-breaker testing**

**(SoC)**

**Awaiting results of tie-breaker test**

Positive result

Negative result

ART does not start within 60 days

Risk of transmission or mortality^2^

ART starts after 60 days

ART does not start within 60 days

ART starts within 60 days

Blood samples are collected at the UHCs and RHCs and tested onsite using the PoC platform. Results are available and provided to the caregiver on the same day. When a positive result is received, a second sample is collected and tested onsite using the PoC platform (ART counseling may commence after the initial positive result). If the confirmatory PoC test is positive, the infant initiates ART immediately. If the confirmatory PoC test is negative, a DBS card is collected and transported to the central lab for testing according to the SoC.If the tie-breaker test is positive, ART is initiated immediately. Due to delays in sample transportation, processing, and caregiver notification, not all caregivers receive timely results.

*Figure 4: PoC+SoC algorithm, Point-of-care test as the initial test and standard-of-care as the confirmatory test*

Enter testing population

**Testing**

**(PoC)**

Positive result

Negative result

ART starts within

60 days

ART doesn’t start within 60 days

Risk of transmission and mortality

Lost before next test

Return for next test

Remain on ART (delayed)

Positive result

*Lost before*

*results are*

*returned*

*Results*

*returned*

*quickly*

*Results*

*not returned*

*quickly*

Remain on ART (rapid)

**Awaiting Results**

**of Second Test**

Never starts ART

ART starts after 60 days

Positive result

**Confirmatory testing**

**(SoC)**

Risk of mortality

Negative result

Negative result

Stop ART

Stop ART

Remain on ART

*Negative & lost before 2^n^ test result*

Remain on ART

*Negative & lost before 2^n^ test result*

Blood samples are collected at the UHCs and RHCs and tested onsite using the PoC platform. Results are available and provided to the caregiver on the same day. When a positive result is received, a DBS card is collected and transported to the central lab for confirmatory testing according to the SoC, and ART is initiated immediately.

If the confirmatory test is positive, the caregiver is notified and the infant remains on ART. If confirmatory test is negative, ART is stopped. Due to delays in sample transportation, processing, and caregiver notification, not all caregivers receive timely results and a proportion of HIV-uninfected infants remain on ART.

*Figure 5: PoC algorithm, Point-of-care test as the initial test without any confirmatory testing.*

Enter testing population

**Testing**

**(PoC)**

Positive result

Negative result

ART starts within

60 days

ART doesn’t start within 60 days

Risk of transmission and mortality

Lost before next test

Return for the next test

Never starts ART

ART starts after 60 days

Risk of transmission and mortality

Blood samples are collected at the UHCs and RHCs and tested onsite using the PoC platform. Results are available and provided to the caregiver on the same day. When a positive result is received, ART is initiated immediately. No confirmatory testing is performed.

While confirmatory testing is currently recommended by the World Health Organization, ART initiation based on a single test may occur in practice. This algorithm was therefore included to understand the impact of testing without confirmation on health and economic outcomes.

1. **Standard of care and point-of-care implementation models**

**Standard of care testing:**

In Zambia, the standard of care for EID is PCR testing at a central lab. In Southern Province, three labs were available or planned by end of the study in Livingstone, Choma, and Mazabuka.

In 2016, the Livingstone Central Hospital lab was performing all EID tests in Southern Province. That year, they received 11,327 DBS from clinics in the province (Figure 6). Assuming that infants were tested on average 1.5 times, we assumed an annual cohort of 7,500 infants requiring testing.

**Point-of-care testing:**

Three implementation models for scaling up PoC testing were considered for the same annual cohort of infants. The models were developed based on the assumption that resources for implementing PoC testing are limited and that PoC platforms will not be placed in every health facility that may see HIV-exposed infants. For the models, decisions about where to place PoC platforms were made based on the volume of samples collected for EID testing (Figure 6).

*Figure 6: PoC site selection by number of tests performed for EID. Source: Ministry of Health program data on the number of tests received by the central labs from healthcare facilities in Southern Province in 2016*


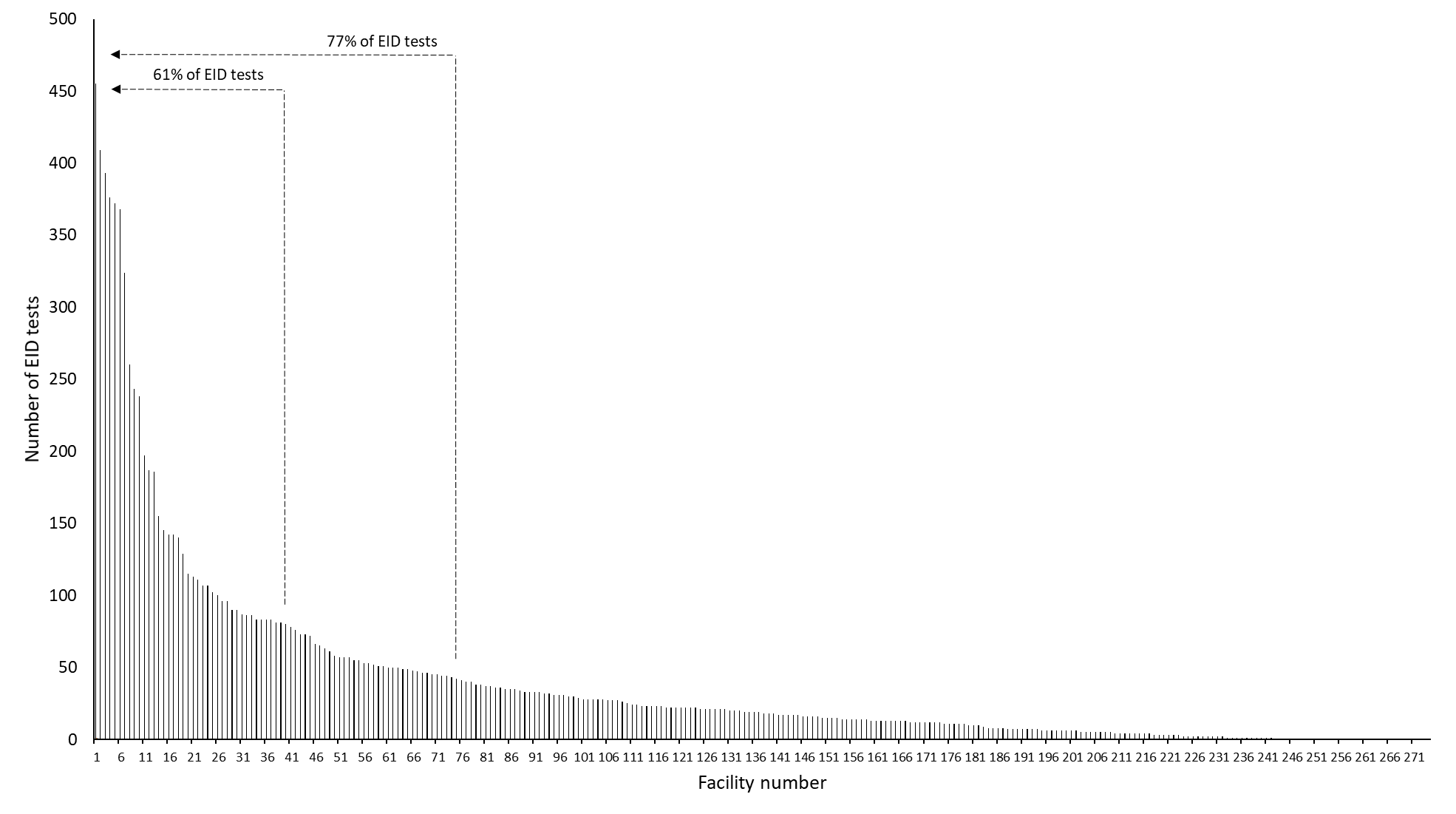


1. In the **primary implementation model,** a limited number of facilities were selected to perform PoC testing based on their volume of samples collected for EID testing (Figure 7). Facilities with a minimum average of 1.5 samples collected per week were selected. Based on programmatic data from 2016 (Figure 6), this resulted in 40 facilities eligible to perform PoC testing, covering 61% of the HIV-exposed infant population. HIV-exposed infants from all other facilities who required EID testing were assumed to be referred to these 40 facilities for testing. We assumed that there were no additional programmatic costs related to referrals and that no children were lost to follow-up for testing due to referral to testing facilities. The costs and epidemiologic inputs associated with the primary implementation model are presented in Supplementary Table 1.

*Figure 7: Primary implementation model*

**Primary**

**Point-of-care testing**

40 PoC platforms

100% of testing by PoC

7,500 children annually

Total health outcomes & costs of PoC testing

1. In the **expanded access implementation model,** a larger number of facilities were selected to perform PoC testing based on their volume of samples collected for EID testing (Figure 8). Facilities with a minimum average of 3.5 samples collected per month were selected. Based on programmatic data from 2016 (Figure 6), this resulted in 74 facilities eligible to perform PoC testing, covering 77% of the HIV-exposed infant population. The remaining 23% of the population was assumed to be tested under the SoC.

The epidemiological inputs for this models were the same as for the primary implementation model. The cost inputs were updated to reflect the proportion of EID tests performed with the SoC (23%) on the SoC’s utilization rate: it was updated from 15% to 3%.

*Figure 8: Expanded implementation model*

**Expanded**

**Point-of-care testing**

74 PoC platforms

77% of testing by PoC

5,775 children annually

Total health outcomes & costs of PoC testing

**Standard-of-care testing**

3 central laboratories

23% of tests by SoC

1,725 children annually

Total health outcomes & costs of SoC testing

1. In the **hub-and-spoke model**, the 40 facilities from the primary implementation model serve as PoC testing hubs (Figure 9). For the 61% of HIV-exposed infants served by the hubs, testing was considered point-of-care, with test results available on the same day. For the remaining 39% of the population, testing was considered near point-of-care, with DBS cards transported from ‘spoke’ facilities to the hubs and test results transported back with the goal of returning to mothers within four-weeks of sample collection. As the m-PIMA is not currently approved for use with DBS cards, this model was only considered for GeneXpert.

The epidemiological inputs were updated to reflect the effect of the delay in receiving results back from the hubs: the probability of starting ART within 60 days was assumed to decrease from 90% to 75% and the probability of starting ART by 12 months of age from 94% to 79%. Transportation costs related to the transport of DBS to the PoC testing hubs were added based on the values used in the SoC algorithm: the utilization rate was decreased to reflect the proportion of EID tests affected (39%) from 15% to 6%.

*Figure 9: Hub-and-spoke implementation model*

**Hub-and-spoke**

**Point-of-care testing “hubs”**

40 PoC platforms

61% of tests by PoC

5,775 children annually

Adjusted total health outcomes & costs of PoC testing

**Point-of-care testing “spokes”**

39% of tests by ‘near’ PoC with transport of DBS to the hubs for testing

1,725 children annually

1. **Model parameters**

All parameters were informed by the literature and from studies conducted in Southern Province, Zambia (see section 4) and represent the best available data at the time the study was conducted. Where possible, the ranges were informed by the range of estimates observed in published studies. However, for many parameters only a few estimates were available and a reasonable range was assumed.

HIV transmission parameters at the different time points were estimated from the literature, including both trials and cohort studies.

Mortality estimates were drawn from a landmark publication following the outcomes of HIV-infected infants in sub-Saharan Africa before ART was widely available in those settings [1]. See below for more information about HIV-related mortality estimation methods.

Three studies had evaluated PoC testing at the time of analysis [2-4]. The estimates of ART initiation within 60 days and overall from these studies were used to inform the range of estimates used in the sensitivity analysis.

PoC assay characteristics in terms of sensitivity and specificity were taken from the WHO technical update [5], which represented a summary from the laboratory and clinical studies of POC assays performed up to the date of publication.

Economic parameters were primarily derived from the NSEBA study. As part of the NSEBA study, the costs of sample collection and testing for both PoC and the SoC were also collected. Using an ingredient-based costing approach, the operating costs for each instrument were estimated in their specific settings (PCR in a central laboratory, GeneXpert and m-PIMA in clinics). Cost data were obtained from Livingstone Central Hospital for lab-based testing in 2016 and from vendor invoices and administrative data for GeneXpert and m-PIMA in 2018 and 2019. The lifespan of the instruments, estimated at 5 years, was based on details provided by the manufacturer. For the SoC, additional cost estimates for freight and installation of the instruments were based on estimates provided by the Clinton Health Access Initiative (CHAI). See section 5 for more information on cost estimation methods and cost estimates.

**HIV-related mortality estimation methods**

This model considers health outcomes from birth to 12 months of age. Over this period, excess mortality among HIV-infected infants prior to ART initiation was simulated assuming an age-dependent mortality function. This age-dependent function was used to account for variable delays in ART initiation and loss to follow-up associated with each testing algorithm. Parameters were derived from survival functions of untreated pediatric HIV cohorts in sub-Saharan Africa between 1999-2004, when ART was not routinely available [1]. Excess HIV-related mortality parameters were calculated as the difference in survival functions between HIV-infected and HIV-uninfected populations at various time points.

*Mortality after testing at birth and six weeks of age*

HIV-infected infants lost to follow-up following a birth or 6-week test experienced a risk of mortality estimated from untreated HIV between birth or 6 weeks and 12 months of age. HIV-infected infants who tested negative at birth or 6 weeks of age (due to false negative results) experienced a risk of mortality from untreated HIV between that age and the age of their next test (i.e. between birth and 6 weeks of age; between 6 weeks and 6 months of age).

HIV-infected infants who tested positive at birth or 6 weeks of age were divided based on the proportion likely to initiate ART rapidly (within 60 days). HIV-infected infants who initiated ART rapidly did not experience risk of HIV-related mortality. HIV-infected infants who tested positive but did not initiate ART rapidly could initiate ART at or before 12 months of age; they were assumed to experience a risk of HIV-related mortality until 12 months of age.

HIV-uninfected infants who were lost to follow-up at birth or 6 weeks experienced a risk of transmission until 12 months of age; infants who became infected over this time experienced a reduced risk of HIV-related mortality between birth or 6 weeks and 12 months of age, informed by untreated HIV mortality estimates among infants who became infected at/after birth or 6 weeks of age.

*Mortality after testing at six months of age*

HIV-infected infants who were lost to follow-up following a 6-month test or who tested negative (due to false negative results) experienced a risk of HIV-related mortality between 6 and 12 months of age.

HIV-infected infants who tested positive at 6 months and did not initiate ART rapidly could initiate ART at or before 12 months of age; they were assumed to experience a risk of HIV-related mortality until 12 months of age. As nucleic acid-based testing did not occur after 6 months in any testing algorithm, we did not incorporate a risk of HIV transmission or HIV-related mortality for infants who were not lost to follow-up and remained HIV-uninfected by 6 months of age.

1. **Early Infant Diagnosis (EID) and Novel Screening for Exposed Babies (NSEBA) studies**

The EID study was a cross-sectional study conducted from 2013 to 2015 at Macha Hospital in Choma District, Southern Province [6]. All infants attending the HIV clinic for EID testing were eligible for enrollment. At enrollment, a questionnaire was administered. When the results of testing were returned, they were documented for the study. As children could have attended the HIV clinic for EID multiple times during the study period, they could be enrolled in the study multiple times. Enrollments for the same child were documented and linked together.

The NSEBA study was a prospective cohort study of HIV-exposed infants conducted at Macha Hospital and four surrounding rural health centers (RHCs) from 2016 to 2019 [7]. HIV-exposed infants born at the sites or attending the sites for EID testing were eligible for enrollment and followed throughout the testing period until post-weaning. At each visit, the mother was administered a questionnaire and a blood sample was collected for EID testing. In October 2018, GeneXpert instruments were implemented in a hub-and-spoke model as part of the study, with Macha Hospital and one of the RHCs serving as hubs. All infants attending the sites and enrolled in the cohort study were eligible to have their samples tested with GeneXpert and results were returned to the mother and clinician for use in clinical care.

*Figure 10. Hub-and-spoke model for the NSEBA study evaluation of GeneXpert*

1. **Cost calculations**

We used an ingredient-based approach to identify and compile cost data from surveys conducted during the NSEBA study [7] and from existing data provided by Clinton Health Access Initiative (CHAI). Costs from the NSEBA study were collected for the quantities used for the study. For simplicity, we assumed that the listed price of any item will not change if items are bulk ordered for testing at the provincial level.

All costs are reported in 2018 USD ($). Costs collected in 2016 for the NSEBA study were corrected to 2018 USD based on an average annual inflation of 8% (16.64% 2-years cumulative) [8].The detailed costs below are presented for the primary implementation model, unless another model (expanded access or hub-and-spoke) is specified. For clarity, those costs are presented in red.

***Capital costs***

*Equation 1: Capital costs calculation*

$$Capital Cost=\sum\left( Cost of {object}_{i} over 5 years\times Quantity of {object}_{i}\times\frac{No. of EID performed}{No. of EID+ Viral load tests performed}\times Utilization rate of {object}_{i} \right)$$

*Capital costs were defined as the program costs to perform any tests; they included:*

1. Cost of purchasing all platforms, which includes service and maintenance, freight and installation, insurance, and other costs over the lifespan of the platform, discounted based on the model time horizon (5 years) and the expected lifespan of the platform.
2. Cost of purchasing the vehicle(s) needed to pick up the drawn blood sample from healthcare facilities, discounted based on the model time horizon (5 years) and the expected lifespan of the vehicle.
3. Cost of the initial training for the medical staff.
4. Cost of communication or delivery systems from the central laboratory to the healthcare facility where the blood sample was collected (SoC only), discounted based on the model time horizon (5 years) and the expected lifespan of the platform.

*Capital costs did not include:*

1. Cost of purchasing the vehicle(s) needed to deliver the necessary supply to healthcare facilities, discounted based on the model time horizon and the expected lifespan of the vehicle. Supplying the facilities is done opportunistically and cannot be quantified formally.
2. Cost of overhead in the healthcare facilities (electricity, water, maintenance, telephone, diesel for generators, oil/paraffin, cleaning, etc.). Data on this cost for the facilities under study were unavailable.

*Capital costs for SoC*

| **Costs included in the capital cost** | **Description (source)** | **Final value** Formula |
| --- | --- | --- |
| *Cost per laboratory platform* | Roche Cobas Ampliprep Taqman (NSEBA) | **$173,423** |
| *Cost of maintenance per platform* | Maintenance included in platform cost (NSEBA) | **N/A** |
| *Cost of setup per platform* | Freight (CHAI, UNICEF Supply Division, applied to all commodities: 8% of $150,000) | **$12,000** |
| *Cost of insurance per platform* | Insurance (CHAI, UNICEF Supply Division, applied to all commodities: 2.5% of $150,000) | **$3,750** |
| *Other costs per platform* | Pre-delivery inspection, UNICEF handling fee, CSD (CHAI, UNICEF Supply Division, applied to all commodities: 1% of $150,000) | **$1,500** |
| *Lifespan of platform* | Expected lifespan of platform (NSEBA) | **60 months** |
| *Quantity of platforms* | Number of central laboratories in the program | **3 platforms** |
| *Utilization rate of platform* | Utilization rate over the time horizon of 12 months of 0.15: with 13,342 EID samples and 76,833 HIV viral load samples tested at the Livingstone Central Hospital (LCH) lab (NSEBA) | **0.15** = 13,342 / (13,342 + 76,833) |
|  |  |  |
| **Medical staff training** | | |
| *Cost of training* | Training of laboratory staff, sum of the one-time training provided to the 2 bio technicians at the LCH lab ($4,469.89). (NSEBA 2016 corrected for 2018). | **$8,121.51** = [$2,493 + $4,469.89] * 1.1664 |
| *Quantity of trainings* | Number of trainings for the PCR platform(s) in the program based on the number of locations | **3 trainings** |
| *Utilization rate of trainings* | Training is not shared with other programs (NSEBA) | **1** |
|  |  |  |
| **Vehicle 1 (rural)** |  |  |
| *Cost of vehicle for rural outreach* | Vehicle to transport the DBS back to the hospital: Sport Utility Vehicle (NSEBA 2016 corrected for 2018) | **$68,818** = $59,000 * 1.1664 |
| *Lifespan of vehicle* | Expected lifespan of vehicle used for transportation (NSEBA) | **84 months** |
| *Quantity of vehicles* | Number of vehicle(s) in the program – 1 for the province (NSEBA) | **1 vehicle** |
| *Utilization rate of vehicle* | The vehicle is used 5 days per week for transporting samples. Samples transported include EID, HIV viral load and tuberculosis – assume EID samples account for 15% of all samples. (NSEBA) | **0.15** |
|  |  |  |
| **Vehicle 2 (urban)** |  |  |
| *Cost of vehicle for urban delivery* | Vehicle to transport the DBS back to the hospital: motorcycle (NSEBA 2016 corrected for 2018) | **$2,333** = $2,000 * 1.1664 |
| *Lifespan of vehicle* | Expected lifespan of vehicle used for transportation (NSEBA) | **60 months** |
| *Quantity of vehicles* | Number of vehicle(s) in the program – 1 per district (NSEBA) | **13 vehicles** |
| *Utilization rate of vehicle* | Proportion of EID samples among all samples collected: 2 days per work week for sample collection and assume that EID samples account for 50% of all samples (NSEBA) | **0.2** = 0.5 * (2 / 5) |
|  |  |  |
| **SMS platform** |  |  |
| *Cost of communication platform* | Cost of SMS machine (NSEBA 2016 corrected for 2018) | **$2,100** = $1,800 * 1.1664 |
| *Lifespan of platform* | Expected lifespan of SMS machine (NSEBA) | **60 months** |
| *Quantity of platforms* | Number of SMS machine(s) in the program – 1 per lab (NSEBA) | **3 machines** |
| *Utilization rate of platform* | Instrument is not shared with other programs (NSEBA) | **1** |

DBS: dried blood spot cards; EID: early infant diagnosis; LCH: Livingstone Central Hospital, SMS: short message service

*Capital cost for CEPHEID GeneXpert IV (PoC)*

| **Costs included in the capital cost** | **Description (source)** | **Final value** Formula |
| --- | --- | --- |
| *Cost per platform* | GeneXpert IV with Desktop PC (NSEBA) | **$17,000** |
| *Cost of maintenance per platform** | 3 Year Warranty Extension (Optional), without travel and Accommodation (NSEBA) | **$6,840** |
| *Cost of setup per platform* | Handling Charges (Bank, Air Freight etc) + Installation & Training + averaged cost of upgrading laboratory in 28 of the 40 UHC and RHC ($2,500 * 28/40 = $1,750) (NSEBA) | **$4,800** = $1,850 + $1,200 + $1,750 |
| *Cost of setup per platform for the expanded access model* | Handling Charges (Bank, Air Freight etc.) + Installation & Training + averaged cost of upgrading laboratory in 59 of the 74 UHC and RHC ($2,500 * 59/74 = $1,993) (NSEBA) | **$5,043** = $1,850 + $1,200 + $1,993 |
| *Cost of insurance per platform* | Insurance included in warranty / maintenance (NSEBA) | **N/A** |
| *Cost of administration per platform* | Team did not purchase the third-party connectivity package (NSEBA) | **$0** |
| *Other costs per platform* | GeneXpert Check ($450), UPS ($790) and printer ($250) (NSEBA) | **$1,490** = $450 + $790 + $250 |
| *Lifespan of platform* | Expected lifespan of platform | **84 months** |
| *Quantity of platforms* | Number of GeneXpert platforms | **40 platforms** |
| *Quantity of platforms for the expanded model* | Number of GeneXpert platforms for the expanded access model | **74 platforms** |
| *Utilization rate of platform* | Non-integrated: 100% dedicated to EID  Integrated: GeneXpert platform is shared for HIV viral load and tuberculosis testing. | **1** (non-integrated) **0.10** (integrated) |
|  |  |  |
| **CEPHEID GeneXpert IV training** | | |
| *Cost of training* | Training included in the cost of setup (NSEBA) | **N/A** |
|  |  |  |
| **Vehicle 1 (rural) for the hub-and-spoke model** | | |
| *Cost of vehicle for rural outreach* | Vehicle to transport the DBS back to the hub (NSEBA 2016 corrected for 2018). | **$68,818** = $59,000 * 1.1664 |
| *Lifespan of vehicle* | Expected lifespan of vehicle used for transportation (NSEBA) | **84 months** |
| *Quantity of vehicles* | Number of vehicle(s) in the program – 1 for the province (NSEBA) | **1 vehicle** |
| *Utilization rate of vehicle* | The vehicle is used 5 days per week for transporting samples. Samples transported include EID, HIV viral and tuberculosis– assume EID samples account for 15% of all samples, adjusted for 40% of facilities (NSEBA) | **0.06** = 0.15 * 0.40 |
|  |  |  |
| **Vehicle 2 (urban) for the hub-and-spoke model** | | |
| *Cost of vehicle for urban delivery* | Vehicle to transport the DBS back to the hub: motorcycle (NSEBA 2016 corrected for 2018) | **$2,333** = $2,000 * 1.1664 |
| *Lifespan of vehicle* | Expected lifespan of vehicle used for transportation in urban areas (NSEBA) | **60 months** |
| *Quantity of vehicles* | Number of vehicle(s) in the program – 1 per district (NSEBA) | **13 vehicles** |
| *Utilization rate of vehicle* | Proportion of EID samples among all samples collected: 2 days per work week for sample collection and assume that EID samples account for 50% of all samples, adjusted for 40% of facilities (NSEBA) | **0.2** = 0.5 * (2 / 5) * 0.40 |

* CHAI reported maintenance costs of $250 per repair for the remaining lifespan of the platform beyond the warranty; however, there is no estimation of the number of repairs needed for the lifespan of the platform. We excluded this value.

*Capital cost for Abbott m-PIMA (PoC)*

| **Costs included in the capital cost** | **Description (source)** | **Final value** Formula |
| --- | --- | --- |
| *Cost per platform* | Cost ($19,875) includes shipping and handling up to Lusaka and 2-year warranty (NSEBA) | **$19,875** |
| *Cost of maintenance per platform* | No usable maintenance costs reported (NSEBA/CHAI) | **Unclear (excluded)** |
| *Cost of setup per platform* | Delivery and installation (NSEBA) | **$167** |
| *Cost of insurance per platform* | No cost reported (NSEBA) | **N/A** |
| *Other costs per platform* | No cost reported (NSEBA) | **N/A** |
| *Lifespan of platform* | Expected lifespan of platform | **60 months** |
| *Quantity of platforms* | Number of m-PIMA platforms | **40 platforms** |
| *Quantity of platforms for the expanded model* | Number of m-PIMA platforms for the expanded access model | **74 platforms** |
| *Utilization rate of platform* | Non-integrated: 100% dedicated to EID  Integrated: m-PIMA can be shared for HIVHIV viral load testing | **1** (non-integrated) **0.15** (integrated) |
|  |  |  |
| **Abbott mPima training** | | |
| *Cost of training* | Training and accommodations for 2 laboratory staff: Free or included with purchase (NSEBA) | **N/A** |

***Recurrent costs***

*Recurrent costs were defined as the costs to perform each test; they included:*

1. Cost of supply needed to perform each test.
2. Cost of the medical staff time spent per test to collect the sample and analyze it, based on the type of staff, their salary and the average time spent per test.
3. Cost of wastage assumed per test, based on the total cost per test times the wastage rate.

To run the model for 5 years, we correct the recurrent costs for inflation to present all the costs in constant USD. Including compound interest, the average inflation rate for 5 years in Zambia is about 50%, or about 4.5% every 6 months. The recurrent costs in the model will be multiplied by 1.045 for every new 6-months cycle.

*Recurrent costs for SoC*

| **Recurrent costs** | **Description (source)** | **Final value** Formula |
| --- | --- | --- |
| *Cost of supply* | Sample collection materials: cost of the kit ($83) for 50 tests (NSEBA 2016 corrected for 2018) | **$1.94** = [$83 / 50] * 1.1664 |
| *Cost of reagents* | Reagents: cost of the kit ($582) for 44 tests (NSEBA 2016 corrected for 2018) | **$15.43** = [$582 / 44] * 1.1664 |
| *Other costs* | CHAI estimate for waste management (CHAI) | **$0.11** |
|  |  |  |
| **Staff time** | | |
| *Cost of staff 1* | Registered Zambian nurse salary to collect specimen: salary per month adjusted for the year, 250 working days, 8 working hours per day (NSEBA 2016 corrected for 2018) | **$4.00** = [$571.7 * 12 / 250 / 8] * 1.1664 |
| *Time spent by staff 1* | Registered Zambian nurse time to collect specimen: 0.17 hour for pre-test counselling, 0.13 hour for sample collection, 0.30 hour for post-test counseling (NSEBA) | **0.6 hour  =** 0.17 + 0.13 + 0.30 |
| *Cost of staff 2* | Biotechnician salary to test specimen: salary for 1 person working at a time per month adjusted for the year, adjusted for 250 working days, 6 working hours per day dedicated to testing (NSEBA 2016 corrected for 2018) | **$20.15** = [$2,159.9 * 12 / 250 / 6] * 1.1664 |
| *Time spent by staff 2* | Biotechnician time to test specimen: 0.35 hour (NSEBA) | **0.35 hour** |
| *Cost of staff 3* | Data associate salary for specimen testing: salary per month adjusted for the year, adjusted for 250 working days, 8 working hours per day (NSEBA 2016 corrected for 2018) | **$17.60** = [$1,099.4 * 2 *12 / 250 / 6] * 1.1664 |
| *Time spent by staff 3* | Data associate time for specimen testing: 0.17 hour (NSEBA) | **0.17 hour** |
| *Cost of staff 4 per test* | Sport Utility Vehicle driver salary ($3,810) at 100% level of work, vehicle maintenance per year ($2,150) adjusted by month, fuel used per week ($303.12) adjusted by month, daily subsistence allowance ($32) adjusted by month, divided by the total number of samples transported monthly for EID (13,342) and HIV viral load (76,833) testing (NSEBA 2016 corrected for 2018) | **$0.89** = [ [ (1 * $3,810) + ($2,150 / 12) + ($303.12 * 4) + ($32 * 4 * 4) ] / ((13,342 + 76,833)/12) ] * 1.1664 |
| *Time spent by staff 4* | Not applicable: cost per test | **1** |
| *Cost of staff 5 per test* | Motorcycle driver salary ($343.70) at 40% level of work (2 days/week), vehicle maintenance per year ($400) adjusted by month, fuel used per week ($35.01) adjusted by month, divided by the total number of samples transported monthly for EID (13,342) and HIV viral load (76,833) testing (NSEBA 2016 corrected for 2018) | **$0.05** = [ [ (0.4 * $343.70) + ($400 / 12) + ($35.01 * 4) ] / ((13,342 + 76,833)/12) ] * 1.1664 |
| *Time spent by staff 5* | Not applicable: cost per test | **1** |
|  |  |  |
| **Other parameters** |  |  |
| *Wastage rate* | If there is any wastage, it is integrated as a negative health outcome (patient never receives results) | **0.01** |

*Recurrent costs for CEPHEID GeneXpert IV (PoC)*

| **Recurrent costs** | **Description (source)** | **Final value** Formula |
| --- | --- | --- |
| *Cost of supply* | Cumulative price of Sarstedt 2mL tubes, cotton roll + spirits, gloves, lancet, biohazard bag adjusted per test (NSEBA) | **$0.455** = 250 / 1000 + 0.1 / 1 + 3.5 / 100 + 3 / 100 + 50 / 200 |
| *Cost of reagents* | 1 Xpert HIV-1 Qual cartridge ($149 for 10 tests) (NSEBA) | **$14.90** |
| *Other costs* | Cumulative cost of collection/storage in a biohazard bag ($0.32), autoclaving labor ($3.00) (assuming 30 mins of labor by a lab tech), electricity for autoclaving ($0.25), labor for incineration ($2.00) (assuming 45 mins of labor by study assistant), and fuel for incineration ($1.50). Total for disposal of 50 cartridges = $7.07 or about $0.14 per cartridge. (NSEBA) | **$0.14** |
|  |  |  |
| **Staff time** | | |
| *Cost of staff 1* | Registered Zambian nurse salary to collect specimen: salary per month adjusted for the year, 250 working days, 8 working hours per day (NSEBA 2016 corrected for 2018) | **$4.00** = [$571.7 * 12 / 250 / 8] * 1.1664 |
| *Time spent by staff 1* | Registered Zambian nurse time to collect specimen: 0.17 hour for pre-test counselling, 0.13 hour for sample collection, 0.3 hour for post-test counseling (NSEBA) | **0.6 hour** = 0.17 + 0.13 + 0.3 |
| *Cost of staff 2* | Lab technologist salary to test the specimen: average salary of a junior ($9,784) & senior ($11,255) staff per year, adjusted for 250 working days, 8 working hours per day (NSEBA 2016 corrected for 2018) | **$12.27** = [ average($11,255;$9,784) / 250 / 8] * 1.1664 |
| *Time spent by staff 2* | Lab technologist salary to test the specimen: hands-on 15 min for analysis (NSEBA) | **0.25 hour** |
| *Cost of staff 3 per test for the hub-and-spoke model* | Sport Utility Vehicle driver salary ($3,810) at 100% level of work, vehicle maintenance per year ($2,150) adjusted by month, fuel used per week ($303.12) adjusted by month, daily subsistence allowance ($32) adjusted by month, divided by the total number of samples transported monthly for EID (13,342) and HIV viral load (76,833) testing (NSEBA 2016 corrected for 2018) | **$0.89** = [ [ (1 * $3,810) + ($2,150 / 12) + ($303.12 * 4) + ($32 * 4 * 4) ] / ((13,342 + 76,833)/12) ] * 1.1664 |
| *Time spent by staff 3 for the hub-and-spoke model* | Not applicable: cost per test | **1** |
| *Cost of staff 4 per test for the hub-and-spoke model* | Motorcycle driver salary ($343.70) at 40% level of work (2 days/week), vehicle maintenance per year ($400) adjusted by month, fuel used per week ($35.01) adjusted by month, divided by the total number of samples transported monthly for EID (13,342) and HIV viral load (76,833) testing (NSEBA 2016 corrected for 2018) | **$0.05** = [ [ (0.4 * $343.70) + ($400 / 12) + ($35.01 * 4) ] / ((13,342 + 76,833)/12) ] * 1.1664 |
| *Time spent by staff 4 for the hub-and-spoke model* | Not applicable: cost per test | **1** |
|  |  |  |
| **Other parameters** |  |  |
| *Wastage rate* | Reported as the error rate (CHAI) | **0.09** |

*Recurrent costs for Abbott m-PIMA (PoC)*

| **Recurrent costs** | **Description (source)** | **Final value** Formula |
| --- | --- | --- |
| *Cost of supply* | Cumulative cost for finger stick sample collection kit and plastic capillaries, adjusted per test (NSEBA) | **$1.3475** = $113.75 / 100 + $210 / 1000 |
| *Cost of reagents* | Alere q HIV ½ Detect test ($1,576.01 for 50 tests) (NSEBA) | **$31.52** = $1,576.01 / 50 |
| *Other costs* | None | **$0** |
|  |  |  |
| **Staff time** | | |
| *Cost of staff 1* | Registered Zambian nurse salary to collect specimen: salary per month adjusted for the year, 250 working days, 8 working hours per day (NSEBA 2016 corrected for 2018) | **$4.00** = [$571.7 * 12 / 250 / 8] * 1.1664 |
| *Time spent by staff 1* | Registered Zambian nurse time to collect specimen: 0.17 hour for pre-test counselling, 0.13 hour for sample collection, 0.3 hours for post-test counseling (NSEBA) | **0.6 hour** = 0.17 + 0.13 + 0.3 |
| *Cost of staff 2* | Registered Zambian nurse salary to collect specimen: salary per month adjusted for the year, 250 working days, 8 working hours per day (NSEBA 2016 corrected for 2018) | **$4.00** = [$571.7 * 12 / 250 / 8] * 1.1664 |
| *Time spent by staff 2* | Registered Zambian nurse salary to test the specimen: hands-on 15 min for analysis (NSEBA) | **0.25 hour** |
|  |  |  |
| **Other parameters** |  |  |
| *Wastage rate* | Reported as the error rate (CHAI) | **0.09** |

1. **Sensitivity analysis**

Sensitivity analyses were conducted using the primary implementation model to evaluate the influence of model parameters on the health outcomes, costs, and ICERs.

*Univariate sensitivity analysis*

A univariate sensitivity analysis was performed by iteratively re-running the model, each time with a single parameter set to a more extreme value than in the primary analysis (Supplemental Table 1). Parameters of interest included the lifespan of the SoC and PoC platforms, sensitivity of the PoC platforms, the time spent by clinic staff on PoC testing, the utilization rate of the PoC platforms, PMTCT coverage, the likelihood of ART initiation with SoC, distribution of infants entering the testing schedule at different ages, and retention of infants in the testing schedule.

*Varying PMTCT coverage*

In both the baseline and worst-case scenarios, we assumed that the proportion of mothers who received drugs for prevention of mother-to-child transmission (PMTCT) would naturally increase over time [9], albeit at different rates. The rates are held constant for simplicity.

The baseline scenario has a starting PMTCT coverage at 93%, increasing by 1% per year over the model horizon (5 years), ending with a coverage of 97% for the 5^th^ year. The worst-case scenario has an initial PMTCT coverage of 73%, increasing by about 3% per year over the model horizon, ending with a coverage of 85%.

The PMTCT coverage of the best-case scenario is held constant at 99%.

*Varying lifespan of the platform*

For the shorter lifespan covering only the period of the warranty, we assumed that any repair to the testing platform within the warranty period would be entirely covered by the warranty without copayment. We also assumed that any repair outside the warranty period would entail the full replacement of the platform.

*Varying testing time with the platform*

From the observations in the NSEBA study, the time spent by healthcare workers to take a blood sample averaged 15 minutes, ranging from 10 to 30 minutes depending on several factors like the child’s behavior, the healthcare worker’s experience, and clinic logistics.

*Multivariate sensitivity analysis*

A multivariate sensitivity analysis was then performed for each PMTCT coverage setting (primary, low, high) to obtain a worst (all parameters of interest set to their lowest/worst value) and best (all parameters of interest set to their highest/best value) case scenario. As the age distribution for entry into testing is linked to PMTCT coverage, the primary, worst and best values for this factor were linked to each PMTCT coverage value (i.e. primary PMTCT=primary testing cohort; best PMTCT=early testing cohort - distribution weighted towards initiating testing at birth; worst PMTCT=late testing cohort - distribution weighted towards initiating testing at 6 weeks and 6 months of age).

**References**

1. Newell ML, Coovadia H, Cortina-Borja M, Rollins N, Gaillard P, Dabis F. Mortality of infected and uninfected infants born to HIV-infected mothers in Africa: a pooled analysis. Lancet. 2004;364(9441):1236-43. PubMed PMID: 15464184.

2. Jani IV, Meggi B, Loquiha O, Tobaiwa O, Mudenyanga C, Zitha A, et al. Effect of point-of-care early infant diagnosis on antiretroviral therapy initiation and retention of patients. AIDS. 2018;32(11):1453-63. Epub 2018/05/11. doi: 10.1097/QAD.0000000000001846. PubMed PMID: 29746301.

3. Mwenda R, Fong Y, Magombo T, Saka E, Midiani D, Mwase C, et al. Significant patient impact observed upon implementation of point-of-care early infant diagnosis technologies in an observational study in Malawi. Clin Infect Dis. 2018;67(5):701-7. Epub 2018/03/01. doi: 10.1093/cid/ciy169. PubMed PMID: 29490026; PubMed Central PMCID: PMCPMC6093992.

4. Bianchi F, Cohn J, Sacks E, Bailey R, Lemaire JF, Machekano R, et al. Evaluation of a routine point-of-care intervention for early infant diagnosis of HIV: an observational study in eight African countries. Lancet HIV. 2019. Epub 2019/04/17. doi: 10.1016/S2352-3018(19)30033-5. PubMed PMID: 30987937.

5. WHO. Information Note - HIV Diagnostics: Novel point-of-care tools for early infant diagnosis of HIV. Geneva, Switzerland: World Health Organization, 2017.

6. Sutcliffe CG, Thuma PE, van Dijk JH, Sinywimaanzi K, Mweetwa S, Hamahuwa M, et al. Use of mobile phones and text messaging to decrease the turnaround time for early infant HIV diagnosis and notification in rural Zambia: an observational study. BMC Pediatr. 2017;17(1):66. doi: 10.1186/s12887-017-0822-z. PubMed PMID: 28270134; PubMed Central PMCID: PMCPMC5341427.

7. Sutcliffe CG, Mutanga JN, Moyo N, Schue JL, Hamahuwa M, Thuma PE, et al. Acceptability and feasibility of testing for HIV infection at birth and linkage to care in rural and urban Zambia: a cross-sectional study. BMC Infect Dis. 2020;20(1):227. Epub 2020/03/19. doi: 10.1186/s12879-020-4947-6. PubMed PMID: 32183751; PubMed Central PMCID: PMCPMC7079396.

8. World Bank. Consumer price index 2019 [cited 2019 September 20]. Available from: <https://data.worldbank.org/indicator/FP.CPI.TOTL>.

9. UNAIDS. AIDSInfo 2018 [cited 2018 August 13]. Available from: <https://aidsinfo.unaids.org/>.
