# Supplementary material for: The cost-effectiveness of scaling-up rapid point-of-care testing for early infant diagnosis of HIV in southern Zambia
Source: PLoS One. 2021 Mar 9;16(3):e0248217. doi: 10.1371/journal.pone.0248217 (PMC7943017; doi:10.1371/journal.pone.0248217)
Supplement: S1 Table — (DOCX) [file pone.0248217.s003.docx]

**S1 Table: Model inputs**

| **Parameter** | **Value** | **Sensitivity analysis range** | **Source** |
| --- | --- | --- | --- |
| **Annual number of children** | 7,500 |  | NSEBA Study |
| **Proportion of children entering the cohort** |  |  |  |
| At birth | 0.40 | 0.15-0.80 | NSEBA Study |
| At 6 weeks of age | 0.45 | 0.45-0.10 | NSEBA Study |
| At 6 months of age | 0.15 | 0.40-0.10 | NSEBA Study |
| **PMTCT coverage** | 0.93 | 0.73-0.99 | [1] |
| **Risk of mother-to-child transmission of HIV** |  |  |  |
| *Mother received PMTCT* |  |  |  |
| Tested at birth | 0.01 |  | [2, 3] |
| Tested at 6 weeks, given tested negative at birth | 0.01 |  | [4, 5] |
| Tested at 6 weeks, given not tested at birth | 0.02 |  | [6-8] |
| Tested at 6 months, given tested negative at 6 weeks | 0.01 |  | [3, 6] |
| Tested at 6 months, given not tested at birth or 6 weeks | 0.08 |  | EID Study [3, 8] |
| *Mother did not receive PMTCT* |  |  |  |
| Tested at birth | 0.08 |  | [9-17] |
| Tested at 6 weeks, given tested negative at birth^b^ | 0.02 |  | [10, 11, 18] |
| Tested at 6 weeks, given not tested at birth | 0.22 |  | EID Study [6, 8, 11, 12, 14, 17] |
| Tested at 6 months, given tested negative at birth and 6 weeks^b^ | 0.01 |  | [3, 6] |
| Tested at 6 months, given tested negative at 6 weeks (no test at birth)^b^ | 0.02 |  | [6, 18] |
| Tested at 6 months, given not tested at birth or 6 weeks | 0.3 |  | EID Study [8, 17] |
| **Probability of ART initiation** |  |  |  |
| After SoC testing, within 60 days | 0.30 | 0.13-0.43 | NSEBA Study  [19-23] |
| After SoC testing, by 12 months of age | 0.55 | 0.35-0.65 | NSEBA Study  [19-23] |
| After PoC testing, within 60 days | 0.90 |  | [19, 20, 23] |
| After PoC testing, by 12 months of age | 0.94 |  | [19, 20] |
| **Probability of returning for subsequent EID testing** |  |  |  |
| Mother received PMTCT | 0.8 | 0.75-0.85 | NSEBA Study |
| Mother did not receive PMTCT | 0.6 | 0.35-0.85 | NSEBA Study |
| For tie-breaker test a week later after discrepant first and second tests (PoC3 algorithm only) | 0.97 |  | Assumption |
| **Probability of receiving test results** |  |  |  |
| After SoC testing | 0.9 |  | NSEBA Study |
| After PoC testing | 1.0 |  | Assumption |
| **Risk of HIV-related mortality** |  |  |  |
| *Among infants infected at birth* |  |  |  |
| Between birth and 6 weeks of age | 0.012 |  | [24] |
| Between birth and 6 months of age | 0.18 |  | [24] |
| Between birth and 12 months of age | 0.33 |  | [24] |
| *Among infants infected at 6 weeks of age* |  |  |  |
| Between 6 weeks and 6 months of age | 0.17 |  | [24] |
| Between 6 weeks and 12 months of age, among infants first tested at 6 weeks | 0.32 |  | [24] |
| Between infection and 12 months of age, among infants testing negative at birth who acquire HIV by 6 weeks of age | 0.32 |  | [24] |
| *Among infants infected at 6 months of age* |  |  |  |
| Between 6 months and 12 months of age, among infants first tested at 6 months of age | 0.15 |  | [24] |
| Between infection and 12 months of age, among infants uninfected at 6 weeks of age who acquire HIV by 6 months of age | 0.19 |  | [24] |
| **SoC characteristics and costs** |  |  |  |
| Sensitivity | 1 |  | Assumption |
| Specificity | 1 |  | Assumption |
| Cost of instrument (included maintenance) | $173,423.00 |  | NSEBA Study |
| Cost of freight and installation | $12,000.00 |  | CHAI |
| Cost of insurance and warranty | $3,750.00 |  | CHAI |
| Cost of staff training | $8,121.51 |  | NSEBA Study |
| Other costs (included pre-delivery inspection, UNICEF handling fee, UNICEF Commodity Supply Division (applied to all commodities) | $1,500.00 |  | CHAI |
| Lifespan of instrument | 5 years | 7 years | NSEBA study, CHAI |
| Utilization rate | 0.15 |  | NSEBA Study |
| Wastage rate^a^ | 1% |  | NSEBA Study |
| Cost of blood collection supplies | $1.94 |  | NSEBA Study |
| Cost of reagents | $15.43 |  | NSEBA Study |
| Cost of waste management | $0.11 |  | CHAI |
| Communication device for SMS results |  |  |  |
| Cost of device | $2,100 |  | NSEBA Study |
| Lifespan of device | 5 years |  | Assumption |
| Utilization rate | 1 |  | NSEBA Study |
| Cost of staff time to run the test and generate a result |  |  |  |
| Cost per hour of laboratory technician | $20.15 |  | NSEBA Study |
| Cost per hour of data associate | $17.60 |  | NSEBA Study |
| Time spent to run each test | 0.35 hour |  | NSEBA Study |
| Time spent per test by data associate | 0.17 hour |  | NSEBA Study |
| **GeneXpert characteristics and costs** |  |  |  |
| Sensitivity | 0.968 | 0.9268-0.9895 | [25] |
| Specificity | 0.9991 |  | [25] |
| Cost of instrument | $17,000.00 |  | NSEBA Study |
| Cost of maintenance (included insurance and warranty) | $6,840.00 |  | NSEBA Study |
| Cost of freight and installation (included upgrading the facilities and staff training) | $4,800.00 |  | NSEBA Study |
| Other costs (included pre-delivery inspection, UPS and printers) | $1,490.00 |  | NSEBA Study |
| Lifespan of instrument | 7 years | 3 years | NSEBA study, CHAI |
| Utilization rate | 1 | 0.10-1.00 | Assumption |
| Wastage rate^a^ | 9% |  | NSEBA Study, CHAI |
| Cost of blood collection supplies | $0.46 |  | NSEBA Study |
| Cost of reagents | $14.90 |  | NSEBA Study |
| Cost of waste management | $0.14 |  | NSEBA Study |
| Cost of staff time to run the test and generate a result |  |  |  |
| Cost per hour of laboratory technician | $12.27 |  | NSEBA Study |
| Time spent to run each test | 0.25 hour | 0.17-0.5 | NSEBA Study |
| **m-PIMA characteristics and costs** |  |  |  |
| Sensitivity | 0.99 | 0.9645-0.9988 | [25] |
| Specificity | 0.9997 |  | [25] |
| Cost of instrument (included maintenance, warranty and staff training) | $19,875.00 |  | NSEBA Study |
| Cost of freight and installation | $167.00 |  | NSEBA Study |
| Lifespan | 5 years | 2 years | NSEBA study, CHAI |
| Utilization rate | 1 | 0.15-1.00 | Assumption |
| Wastage rate^a^ | 9% |  | CHAI [26, 27] |
| Cost of blood collection supplies | $1.35 |  | NSEBA Study |
| Cost of reagents | $31.52 |  | NSEBA Study |
| Cost of waste management | $0.00 |  | Assumption |
| Cost of staff time to run the test and generate a result |  |  |  |
| Cost per hour of registered nurse | $4.00 |  | NSEBA Study |
| Time spent to run each test | 0.25 hour | 0.17-0.5 | NSEBA Study |
| **Transportation of samples** |  |  |  |
| *Rural areas* |  |  |  |
| Cost of vehicle (SUV) | $68,818.00 |  | NSEBA Study |
| Lifespan of vehicle | 7 years |  | Assumption |
| Utilization rate | 0.15 |  | NSEBA Study |
| Cost per test of driver | $0.89 |  | NSEBA Study |
| *Urban areas* |  |  |  |
| Cost of vehicle (motorcycle) | $2,333.00 |  | NSEBA Study |
| Lifespan of vehicle | 5 years |  | Assumption |
| Utilization rate | 0.2 |  | NSEBA Study |
| Cost per test of driver | $0.05 |  | NSEBA Study |
| **Cost of staff time for blood collection for SoC and PoC** |  |  |  |
| Cost per hour of registered nurse | $4.00 |  | NSEBA Study |
| Time spent per test on sample collection (includes pre-test counseling) | 0.6 hour | 0.5-1.0 | NSEBA Study |

^a^ Proportion of tests that yield an error or invalid result and need to be re-run

^b^  With maternal ART and infant PMTCT initiated at the time of first test

EID: early infant diagnosis; PMTCT: prevention of mother-to-child transmission; PoC: point-of-care; PoC3 algorithm: PoC testing for initial test, PoC for confirmatory test, PoC test for tie-breaker test in the event of a discrepancy between the initial and confirmatory test; SoC: standard of care; SUV: sport utility vehicle; SMS: short message service; UPS: uninterruptable power supply

**References**

1. WHO. Prevention of mother-to-child transmission: Estimates by country Geneva, Switzerland2018 [cited 2018 August 13]. Available from: <http://apps.who.int/gho/data/node.main.627?lang=en>.

2. Shapiro RL, Hughes MD, Ogwu A, Kitch D, Lockman S, Moffat C, et al. Antiretroviral regimens in pregnancy and breast-feeding in Botswana. N Engl J Med. 2010;362(24):2282-94. Epub 2010/06/18. doi: 10.1056/NEJMoa0907736. PubMed PMID: 20554983; PubMed Central PMCID: PMC2999916.

3. Kilewo C, Karlsson K, Ngarina M, Massawe A, Lyamuya E, Swai A, et al. Prevention of mother-to-child transmission of HIV-1 through breastfeeding by treating mothers with triple antiretroviral therapy in Dar es Salaam, Tanzania: the Mitra Plus study. J Acquir Immune Defic Syndr. 2009;52(3):406-16. Epub 2009/09/05. doi: 10.1097/QAI.0b013e3181b323ff. PubMed PMID: 19730269.

4. Chasela CS, Hudgens MG, Jamieson DJ, Kayira D, Hosseinipour MC, Kourtis AP, et al. Maternal or infant antiretroviral drugs to reduce HIV-1 transmission. N Engl J Med. 2010;362(24):2271-81. Epub 2010/06/18. doi: 10.1056/NEJMoa0911486. PubMed PMID: 20554982; PubMed Central PMCID: PMCPMC3440865.

5. Flynn PM, Taha TE, Cababasay M, Fowler MG, Mofenson LM, Owor M, et al. Prevention of HIV-1 transmission through breastfeeding: Efficacy and safety of maternal antiretroviral therapy versus infant nevirapine prophylaxis for duration of breastfeeding in HIV-1-infected women with high CD4 cell count (IMPAACT PROMISE): A randomized, open-label, clinical trial. J Acquir Immune Defic Syndr. 2018;77(4):383-92. Epub 2017/12/15. doi: 10.1097/QAI.0000000000001612. PubMed PMID: 29239901; PubMed Central PMCID: PMCPMC5825265.

6. Dinh TH, Mushavi A, Shiraishi RW, Tippett Barr B, Balachandra S, Shambira G, et al. Impact of timing of antiretroviral treatment and birth weight on mother-to-child human immunodeficiency virus transmission: Findings from an 18-month prospective cohort of a nationally representative sample of mother-infant pairs during the transition from Option A to Option B+ in Zimbabwe. Clin Infect Dis. 2018;66(4):576-85. Epub 2018/02/06. doi: 10.1093/cid/cix820. PubMed PMID: 29401270.

7. Six Week Extended-Dose Nevirapine Study T, Bedri A, Gudetta B, Isehak A, Kumbi S, Lulseged S, et al. Extended-dose nevirapine to 6 weeks of age for infants to prevent HIV transmission via breastfeeding in Ethiopia, India, and Uganda: an analysis of three randomised controlled trials. Lancet. 2008;372(9635):300-13. Epub 2008/07/29. doi: 10.1016/S0140-6736(08)61114-9. PubMed PMID: 18657709.

8. Saounde Temgoua EM, Nkenfou CN, Zoung-Kanyi Bissek AC, Fokam J, Billong SC, Sosso SM, et al. HIV-1 early infant diagnosis is an effective indicator of the prevention of mother-to-child transmission program performance: Experience from Cameroon. Curr HIV Res. 2015;13(4):286-91. Epub 2015/04/08. PubMed PMID: 25845391.

9. De Cock KM, Fowler MG, Mercier E, de Vincenzi I, Saba J, Hoff E, et al. Prevention of mother-to-child HIV transmission in resource-poor countries: translating research into policy and practice. JAMA. 2000;283(9):1175-82. PubMed PMID: 10703780.

10. Kumwenda NI, Hoover DR, Mofenson LM, Thigpen MC, Kafulafula G, Li Q, et al. Extended antiretroviral prophylaxis to reduce breast-milk HIV-1 transmission. N Engl J Med. 2008;359(2):119-29. Epub 2008/06/06. doi: 10.1056/NEJMoa0801941. PubMed PMID: 18525035.

11. Connor EM, Sperling RS, Gelber R, Kiselev P, Scott G, O'Sullivan MJ, et al. Reduction of maternal-infant transmission of human immunodeficiency virus type 1 with zidovudine treatment. Pediatric AIDS Clinical Trials Group Protocol 076 Study Group. N Engl J Med. 1994;331(18):1173-80. Epub 1994/11/03. doi: 10.1056/NEJM199411033311801. PubMed PMID: 7935654.

12. Guay LA, Musoke P, Fleming T, Bagenda D, Allen M, Nakabiito C, et al. Intrapartum and neonatal single-dose nevirapine compared with zidovudine for prevention of mother-to-child transmission of HIV-1 in Kampala, Uganda: HIVNET 012 randomised trial. Lancet. 1999;354(9181):795-802. Epub 1999/09/15. doi: 10.1016/S0140-6736(99)80008-7. PubMed PMID: 10485720.

13. Shaffer N, Chuachoowong R, Mock PA, Bhadrakom C, Siriwasin W, Young NL, et al. Short-course zidovudine for perinatal HIV-1 transmission in Bangkok, Thailand: a randomised controlled trial. Bangkok Collaborative Perinatal HIV Transmission Study Group. Lancet. 1999;353(9155):773-80. Epub 1999/08/25. doi: 10.1016/s0140-6736(98)10411-7. PubMed PMID: 10459957.

14. Wiktor SZ, Ekpini E, Karon JM, Nkengasong J, Maurice C, Severin ST, et al. Short-course oral zidovudine for prevention of mother-to-child transmission of HIV-1 in Abidjan, Cote d'Ivoire: a randomised trial. Lancet. 1999;353(9155):781-5. Epub 1999/08/25. doi: 10.1016/S0140-6736(98)10412-9. PubMed PMID: 10459958.

15. Bertolli J, St Louis ME, Simonds RJ, Nieburg P, Kamenga M, Brown C, et al. Estimating the timing of mother-to-child transmission of human immunodeficiency virus in a breast-feeding population in Kinshasa, Zaire. J Infect Dis. 1996;174(4):722-6. Epub 1996/10/01. doi: 10.1093/infdis/174.4.722. PubMed PMID: 8843208.

16. Gray GE, Urban M, Chersich MF, Bolton C, van Niekerk R, Violari A, et al. A randomized trial of two postexposure prophylaxis regimens to reduce mother-to-child HIV-1 transmission in infants of untreated mothers. AIDS. 2005;19(12):1289-97. Epub 2005/07/30. doi: 10.1097/01.aids.0000180100.42770.a7. PubMed PMID: 16052084.

17. Dabis F, Msellati P, Meda N, Welffens-Ekra C, You B, Manigart O, et al. 6-month efficacy, tolerance, and acceptability of a short regimen of oral zidovudine to reduce vertical transmission of HIV in breastfed children in Cote d'Ivoire and Burkina Faso: a double-blind placebo-controlled multicentre trial. DITRAME Study Group. DIminution de la Transmission Mere-Enfant. Lancet. 1999;353(9155):786-92. Epub 1999/08/25. doi: 10.1016/s0140-6736(98)11046-2. PubMed PMID: 10459959.

18. Taha TE, Li Q, Hoover DR, Mipando L, Nkanaunena K, Thigpen MC, et al. Postexposure prophylaxis of breastfeeding HIV-exposed infants with antiretroviral drugs to age 14 weeks: updated efficacy results of the PEPI-Malawi trial. J Acquir Immune Defic Syndr. 2011;57(4):319-25. Epub 2011/03/23. doi: 10.1097/QAI.0b013e318217877a. PubMed PMID: 21423025.

19. Jani IV, Meggi B, Loquiha O, Tobaiwa O, Mudenyanga C, Zitha A, et al. Effect of point-of-care early infant diagnosis on antiretroviral therapy initiation and retention of patients. AIDS. 2018;32(11):1453-63. Epub 2018/05/11. doi: 10.1097/QAD.0000000000001846. PubMed PMID: 29746301.

20. Mwenda R, Fong Y, Magombo T, Saka E, Midiani D, Mwase C, et al. Significant patient impact observed upon implementation of point-of-care early infant diagnosis technologies in an observational study in Malawi. Clin Infect Dis. 2018;67(5):701-7. Epub 2018/03/01. doi: 10.1093/cid/ciy169. PubMed PMID: 29490026; PubMed Central PMCID: PMCPMC6093992.

21. Sutcliffe CG, Thuma PE, van Dijk JH, Sinywimaanzi K, Mweetwa S, Hamahuwa M, et al. Use of mobile phones and text messaging to decrease the turnaround time for early infant HIV diagnosis and notification in rural Zambia: an observational study. BMC pediatrics. 2017;17(1):66. doi: 10.1186/s12887-017-0822-z. PubMed PMID: 28270134; PubMed Central PMCID: PMCPMC5341427.

22. Sutcliffe CG, Mutanga JN, Moyo N, Schue JL, Hamahuwa M, Thuma PE, et al. Acceptability and feasibility of testing for HIV infection at birth and linkage to care in rural and urban Zambia: a cross-sectional study. BMC Infect Dis. 2020;20(1):227. Epub 2020/03/19. doi: 10.1186/s12879-020-4947-6. PubMed PMID: 32183751; PubMed Central PMCID: PMCPMC7079396.

23. Bianchi F, Cohn J, Sacks E, Bailey R, Lemaire JF, Machekano R, et al. Evaluation of a routine point-of-care intervention for early infant diagnosis of HIV: an observational study in eight African countries. Lancet HIV. 2019. Epub 2019/04/17. doi: 10.1016/S2352-3018(19)30033-5. PubMed PMID: 30987937.

24. Newell ML, Coovadia H, Cortina-Borja M, Rollins N, Gaillard P, Dabis F. Mortality of infected and uninfected infants born to HIV-infected mothers in Africa: a pooled analysis. Lancet. 2004;364(9441):1236-43. PubMed PMID: 15464184.

25. WHO. Information Note - HIV Diagnostics: Novel point-of-care tools for early infant diagnosis of HIV. Geneva, Switzerland: World Health Organization, 2017.

26. Dunning L, Kroon M, Hsiao NY, Myer L. Field evaluation of HIV point-of-care testing for early infant diagnosis in Cape Town, South Africa. PLoS One. 2017;12(12):e0189226. Epub 2017/12/21. doi: 10.1371/journal.pone.0189226. PubMed PMID: 29261707; PubMed Central PMCID: PMCPMC5738050.

27. Meggi B, Vojnov L, Mabunda N, Vubil A, Zitha A, Tobaiwa O, et al. Performance of point-of-care birth HIV testing in primary health care clinics: An observational cohort study. PLoS One. 2018;13(6):e0198344. Epub 2018/06/19. doi: 10.1371/journal.pone.0198344. PubMed PMID: 29912987; PubMed Central PMCID: PMCPMC6005575.
