# Supplementary material for: The cost-effectiveness of scaling-up rapid point-of-care testing for early infant diagnosis of HIV in southern Zambia
Source: PLoS One. 2021 Mar 9;16(3):e0248217. doi: 10.1371/journal.pone.0248217 (PMC7943017; doi:10.1371/journal.pone.0248217)
Supplement: S2 Table — (DOCX) [file pone.0248217.s004.docx]

**S2 Table. Health outcomes and costs by testing platform and algorithm for the primary implementation model**

|  | **SoC** | **GeneXpert** | | | | **m-PIMA** | | | |
| --- | --- | --- | --- | --- | --- | --- | --- | --- | --- |
|  |  | **PoC3** | **PoC2+SoC** | **PoC+SoC** | **PoC** | **PoC3** | **PoC2+SoC** | **PoC+SoC** | **PoC** |
| **MODEL OUTPUTS** |  |  |  |  |  |  |  |  |  |
| Number of HIV-infected children | 1,692 | 1,692 | 1,692 | 1,692 | 1,692 | 1,692 | 1,692 | 1,692 | 1,692 |
| Number of HIV-infected children diagnosed with testing | 1,567 | 1,530 | 1,533 | 1,532 | 1,532 | 1,556 | 1,556 | 1,556 | 1,556 |
| Number of SoC tests | 72,207 | 0 | 112 | 1,499 | 0 | 0 | 36 | 1,483 | 0 |
| Number of PoC tests | 0 | 73,065 | 72,957 | 71,323 | 71,323 | 72,963 | 72,928 | 71,337 | 71,337 |
| **HEALTH OUTCOMES** |  |  |  |  |  |  |  |  |  |
| **ART within 60 days** |  |  |  |  |  |  |  |  |  |
| Number | 470 | 1,377 | 1,350 | 1,379 | 1,379 | 1,400 | 1,392 | 1,401 | 1,401 |
| % | 27.8 | 81.4 | 79.8 | 81.5 | 81.5 | 82.8 | 82.2 | 82.8 | 82.8 |
| Additional compared to SoC | n/a | 907 | 880 | 909 | 909 | 930 | 922 | 931 | 931 |
| **Treated by 12 months** |  |  |  |  |  |  |  |  |  |
| Number | 862 | 1,438 | 1,422 | 1,440 | 1,440 | 1,463 | 1,457 | 1,463 | 1,463 |
| % | 50.9 | 85.0 | 84.0 | 85.1 | 85.1 | 86.4 | 86.1 | 86.5 | 86.5 |
| Additional compared to SoC | n/a | 576 | 560 | 578 | 578 | 601 | 595 | 601 | 601 |
| **Deaths** |  |  |  |  |  |  |  |  |  |
| Number | 307 | 71 | 78 | 70 | 70 | 65 | 67 | 65 | 65 |
| % | 18.1 | 4.2 | 4.6 | 4.2 | 4.2 | 3.8 | 4.0 | 3.8 | 3.8 |
| Averted compared to SoC | n/a | 236 | 229 | 237 | 237 | 242 | 240 | 242 | 242 |
| **False diagnoses** |  |  |  |  |  |  |  |  |  |
| % among children on ART | 0.00 | 0.01 | 0.00 | 0.41 | 3.93 | 0.00 | 0.00 | 0.13 | 1.33 |
| **COSTS** |  |  |  |  |  |  |  |  |  |
| Capital costs | $129,907 | $860,857 | $898,037 | $898,037 | $860,857 | $801,680 | $838,860 | $838,860 | $801,680 |
| Recurrent costs | $2,749,175 | $2,039,358 | $2,040,591 | $2,047,731 | $1,991,037 | $3,522,788 | $3,522,484 | $3,500,897 | $3,444,847 |
| Total program costs | $2,879,081 | $2,900,215 | $2,938,627 | $2,945,768 | $2,851,894 | $4,324,468 | $4,361,343 | $4,339,757 | $4,246,527 |
| **ICERs ($ per additional child)** |  |  |  |  |  |  |  |  |  |
| ART within 60 days | n/a | $23 | $68 | $73 | -$30 | $1,554 | $1,609 | $1,570 | $1,469 |
| ART by 12 months | n/a | $37 | $106 | $115 | -$47 | $2,406 | $2,491 | $2,430 | $2,275 |
| Deaths averted | n/a | $90 | $261 | $282 | -$115 | $5,976 | $6,188 | $6,036 | $5,651 |

ART: antiretroviral therapy; ICER: incremental cost effectiveness ratio; n/a: not applicable; PoC: point-of-care; SoC: standard of care

Note: Four algorithms were modeled for PoC testing. All algorithms started with an initial PoC test performed at the clinic and then differed on confirmatory testing for positive initial tests. The first and second algorithms assumed that confirmatory testing would be performed at the clinic with a second PoC test, with a tie-breaker test for discrepancies performed at the central laboratory (PoC2+SoC) or with a third PoC test at the clinic (PoC3). In both algorithms, while ART counseling could begin after an initial positive result, ART initiation was assumed to occur after results of the tie-breaker test were available. The third algorithm assumed that confirmatory testing would be performed at the central laboratory (PoC+SoC). ART initiation was assumed after the initial positive test result, with treatment interruption in the event of a negative confirmatory test. The last algorithm did not include confirmatory testing and was based on a single PoC test. (PoC).
