# Supplementary material for: The cost-effectiveness of scaling-up rapid point-of-care testing for early infant diagnosis of HIV in southern Zambia
Source: PLoS One. 2021 Mar 9;16(3):e0248217. doi: 10.1371/journal.pone.0248217 (PMC7943017; doi:10.1371/journal.pone.0248217)
Supplement: S3 Table — (DOCX) [file pone.0248217.s005.docx]

**S3 Table. Sensitivity analysis of intrinsic factors influencing costs**

|  | **Primary analysis** | | | **GeneXpert** | | | | **m-PIMA** | | | |
| --- | --- | --- | --- | --- | --- | --- | --- | --- | --- | --- | --- |
|  | **SoC** | **GeneXpert** | **m-PIMA** | **Sensitivity** | | **Test run time** | | **Sensitivity** | | **Test run time** | |
|  |  |  |  | **Low** | **High** | **Short** | **Long** | **Low** | **High** | **Short** | **Long** |
| **HEALTH OUTCOMES** |  |  |  |  |  |  |  |  |  |  |  |
| **ART within 60 days** |  |  |  |  |  |  |  |  |  |  |  |
| Number | 470 | 1,377 | 1,400 | 1,331 | 1400 | 1,377 | 1,377 | 1,373 | 1409 | 1,400 | 1,400 |
| % | 27.8 | 81.4 | 82.8 | 78.6 | 82.7 | 81.4 | 81.4 | 81.2 | 83.3 | 82.8 | 82.8 |
| Additional compared to SoC | n/a | 907 | 930 | 860 | 930 | 907 | 907 | 903 | 939 | 930 | 930 |
| **Treated by 12 months** |  |  |  |  |  |  |  |  |  |  |  |
| Number | 862 | 1,438 | 1,463 | 1,390 | 1,462 | 1,438 | 1,438 | 1,434 | 1,472 | 1,463 | 1,463 |
| % | 50.9 | 85.0 | 86.4 | 82.1 | 86.4 | 85.0 | 85.0 | 84.8 | 87.0 | 86.4 | 86.4 |
| Additional compared to SoC | n/a | 576 | 601 | 528 | 600 | 576 | 576 | 572 | 610 | 601 | 601 |
| **Deaths** |  |  |  |  |  |  |  |  |  |  |  |
| Number | 307 | 71 | 65 | 84 | 65 | 71 | 71 | 72 | 62 | 65 | 65 |
| % | 18.1 | 4.2 | 3.8 | 4.9 | 3.8 | 4.2 | 4.2 | 4.3 | 3.7 | 3.8 | 3.8 |
| Averted compared to SoC | n/a | 236 | 242 | 223 | 242 | 236 | 236 | 235 | 244 | 242 | 242 |
| **False diagnoses** |  |  |  |  |  |  |  |  |  |  |  |
| % among children on ART | 0.00 | 0.01 | 0.00 | 0.01 | 0.01 | 0.01 | 0.01 | 0.00 | 0.00 | 0.00 | 0.00 |
| **COSTS** |  |  |  |  |  |  |  |  |  |  |  |
| Capital costs | $129,907 | $860,857 | $801,680 | $860,857 | $860,857 | $860,857 | $860,857 | $801,680 | $801,680 | $801,680 | $801,680 |
| Recurrent costs | $2,749,175 | $2,039,358 | $3,522,788 | $2,040,397 | $2,038,797 | $1,943,885 | $2,337,712 | $3,523,938 | $3,522,385 | $3,491,708 | $3,619,915 |
| Total program costs | $2,879,081 | $2,900,215 | $4,324,468 | $2,901,254 | $2,899,654 | $2,804,742 | $3,198,569 | $4,325,618 | $4,324,065 | $4,293,388 | $4,421,595 |
| **ICERs ($ per additional child)** |  |  |  |  |  |  |  |  |  |  |  |
| ART within 60 days | n/a | $23 | $1,554 | $26 | $22 | -$82 | $352 | $1,602 | $1,539 | $1,520 | $1,658 |
| ART by 12 months | n/a | $37 | $2,406 | $42 | $34 | -$129 | $554 | $2,527 | $2,369 | $2,355 | $2,568 |
| Deaths averted | n/a | $90 | $5,976 | $99 | $85 | -$316 | $1,356 | $6,168 | $5,915 | $5,848 | $6,378 |

ART: antiretroviral therapy; ICER: incremental cost effectiveness ratio; n/a: not applicable; PoC: point-of-care; SoC: standard of care

Note: All sensitivity analyses were performed with the PoC3 algorithm (PoC testing for initial test, PoC for confirmatory test, PoC test for tie-breaker test in the event of a discrepancy between the initial and confirmatory test) and primary implementation model. PoC3 represents the results from the primary analysis with the baseline model parameters (see Supplemental Table 1). Sensitivity refers to the sensitivity of the PoC platform: low=0.9268 for GeneXpert and 0.9645 for m-PIMA; high=0.9895 for GeneXpert and 0.9988 for m-PIMA (compared to 0.968 for GeneXpert and 0.99 for m-PIMA in the primary analysis); long and short run time per test refers to the amount of staff time spent for sample collection (including pre-test counseling) and running the PoC test: long=1.0 hours for sample collection and 0.5 hours for running the test; short=0.5 hours for sample collection and 0.17 hours for running the test (compared to 0.6 hours for sample collection and 0.25 hours for running the test in the primary analysis).

**S3 Table. Sensitivity analysis of intrinsic factors influencing costs, continued**

|  | **Primary analysis** | | | **Long lifespan of SoC** | | | **GeneXpert** | | **m-PIMA** | |
| --- | --- | --- | --- | --- | --- | --- | --- | --- | --- | --- |
|  | **SoC** | **GeneXpert** | **m-PIMA** | **SoC** | **GeneXpert** | **m-PIMA** | **Short lifespan** | **Integrated use** | **Short lifespan** | **Integrated use** |
| **HEALTH OUTCOMES** |  |  |  |  |  |  |  |  |  |  |
| **ART within 60 days** |  |  |  |  |  |  |  |  |  |  |
| Number | 470 | 1,377 | 1,400 | 470 | 1,377 | 1,400 | 1,377 | 1,377 | 1,400 | 1,400 |
| % | 27.8 | 81.4 | 82.8 | 27.8 | 81.4 | 82.8 | 81.4 | 81.4 | 82.8 | 82.8 |
| Additional compared to SoC | n/a | 907 | 930 | n/a | 907 | 930 | 907 | 907 | 930 | 930 |
| **Treated by 12 months** |  |  |  |  |  |  |  |  |  |  |
| Number | 862 | 1,438 | 1,463 | 862 | 1,438 | 1,463 | 1,438 | 1,438 | 1,463 | 1,463 |
| % | 50.9 | 85.0 | 86.4 | 50.9 | 85.0 | 86.4 | 85.0 | 85.0 | 86.4 | 86.4 |
| Additional compared to SoC | n/a | 576 | 601 | n/a | 576 | 601 | 576 | 576 | 601 | 601 |
| **Deaths** |  |  |  |  |  |  |  |  |  |  |
| Number | 307 | 71 | 65 | 307 | 71 | 65 | 71 | 71 | 65 | 65 |
| % | 18.1 | 4.2 | 3.8 | 18.1 | 4.2 | 3.8 | 4.2 | 4.2 | 3.8 | 3.8 |
| Averted compared to SoC | n/a | 236 | 242 | n/a | 236 | 242 | 236 | 236 | 242 | 242 |
| **False diagnoses** |  |  |  |  |  |  |  |  |  |  |
| % among children on ART | 0.00 | 0.01 | 0.00 | 0.00 | 0.01 | 0.00 | 0.01 | 0.01 | 0.00 | 0.00 |
| **COSTS** |  |  |  |  |  |  |  |  |  |  |
| Capital costs | $129,907 | $860,857 | $801,680 | $98,430 | $860,857 | $801,680 | $2,008,667 | $86,086 | $2,004,200 | $120,252 |
| Recurrent costs | $2,749,175 | $2,039,358 | $3,522,788 | $2,749,175 | $2,039,358 | $3,522,788 | $2,039,358 | $2,039,358 | $3,522,788 | $3,522,788 |
| Total program costs | $2,879,081 | $2,900,215 | $4,324,468 | $2,847,605 | $2,900,215 | $4,324,468 | $4,048,025 | $2,125,444 | $5,526,988 | $3,643,040 |
| **ICERs ($ per additional child)** |  |  |  |  |  |  |  |  |  |  |
| ART within 60 days | n/a | $23 | $1,554 | n/a | $58 | $1,588 | $1,289 | -$831 | $2,847 | $821 |
| ART by 12 months | n/a | $37 | $2,406 | n/a | $91 | $2,459 | $,2028 | -$1,307 | $4,409 | $1,272 |
| Deaths averted | n/a | $90 | $5,976 | n/a | $223 | $6,107 | $4,963 | -$3,199 | $10,949 | $3,159 |

ART: antiretroviral therapy; ICER: incremental cost effectiveness ratio; n/a: not applicable; PoC: point-of-care; SoC: standard of care

Note: All sensitivity analyses were performed with the PoC3 algorithm (PoC testing for initial test, PoC for confirmatory test, PoC test for tie-breaker test in the event of a discrepancy between the initial and confirmatory test) and primary implementation model. PoC3 represents the results from the primary analysis with the baseline model parameters (see Supplemental Table 1). Long lifespan of SoC refers to the time period covered by the manufacturer’s warranty: 7 years (compared to 5 years in the primary analysis); Short lifespan for the PoC platforms refers to the time period covered by the manufacturer’s warranty: 3 years for GeneXpert and 2 years for m-PIMA (compared to 7 years for GeneXpert and 5 years for m-PIMA in the primary analysis); integrated use refers to use of the PoC instruments across programs (HIV viral load and tuberculosis testing).
