# Supplementary material for: The cost-effectiveness of scaling-up rapid point-of-care testing for early infant diagnosis of HIV in southern Zambia
Source: PLoS One. 2021 Mar 9;16(3):e0248217. doi: 10.1371/journal.pone.0248217 (PMC7943017; doi:10.1371/journal.pone.0248217)
Supplement: S4 Table — (DOCX) [file pone.0248217.s006.docx]

**S4 Table. Sensitivity analysis of health outcomes and costs by PMTCT coverage**

|  | **Primary PMTCT** | | | **Low PMTCT** | | | **High PMTCT** | | |
| --- | --- | --- | --- | --- | --- | --- | --- | --- | --- |
|  | **SoC** | **GeneXpert** | **m-PIMA** | **SoC** | **GeneXpert** | **m-PIMA** | **SoC** | **GeneXpert** | **m-PIMA** |
| **HEALTH OUTCOMES** |  |  |  |  |  |  |  |  |  |
| **ART within 60 days** |  |  |  |  |  |  |  |  |  |
| Number | 470 | 1,377 | 1,400 | 743 | 2,178 | 2,214 | 385 | 1,127 | 1,146 |
| % | 27.8 | 81.4 | 82.8 | 28.2 | 82.7 | 84.1 | 27.5 | 80.6 | 82.0 |
| Additional compared to SoC | n/a | 907 | 930 | n/a | 1,435 | 1,471 | n/a | 742 | 761 |
| **Treated by 12 months** |  |  |  |  |  |  |  |  |  |
| Number | 862 | 1,438 | 1,463 | 1,363 | 2,275 | 2,312 | 705 | 1,177 | 1,197 |
| % | 50.9 | 85.0 | 86.4 | 51.8 | 86.4 | 87.8 | 50.4 | 84.2 | 85.6 |
| Additional compared to SoC | n/a | 576 | 601 | n/a | 912 | 950 | n/a | 472 | 492 |
| **Deaths** |  |  |  |  |  |  |  |  |  |
| Number | 307 | 71 | 65 | 499 | 109 | 98 | 246 | 59 | 54 |
| % | 18.1 | 4.2 | 3.8 | 19.0 | 4.1 | 3.7 | 17.5 | 4.2 | 3.9 |
| Averted compared to SoC | n/a | 236 | 242 | n/a | 390 | 401 | n/a | 187 | 192 |
| **False diagnoses** |  |  |  |  |  |  |  |  |  |
| % among children on ART | 0.00 | 0.01 | 0.00 | 0.00 | 0.01 | 0.00 | 0.00 | 0.02 | 0.00 |
| **COSTS** |  |  |  |  |  |  |  |  |  |
| Capital costs | $129,907 | $860,857 | $801,680 | $129,907 | $860,857 | $801,680 | $129,907 | $860,857 | $801,680 |
| Recurrent costs | $2,749,175 | $2,039,358 | $3,522,788 | $2,687,677 | $2,005,804 | $3,464,299 | $2,768,113 | $2,049,691 | $3,540,799 |
| Total program costs | $2,879,081 | $2,900,215 | $4,324,468 | $2,817,583 | $2,866,661 | $4,265,979 | $2,898,019 | $2,910,548 | $4,342,479 |
| **ICERs ($ per additional child)** |  |  |  |  |  |  |  |  |  |
| ART within 60 days | n/a | $23 | $1,554 | n/a | $34 | $985 | n/a | $17 | $1,897 |
| ART by 12 months | n/a | $37 | $2,406 | n/a | $54 | $1,525 | n/a | $27 | $2,939 |
| Deaths averted | n/a | $90 | $5,976 | n/a | $126 | $5,976 | n/a | $67 | $7,514 |

ART: antiretroviral therapy; ICER: incremental cost effectiveness ratio; n/a: not applicable; PoC: point-of-care; SoC: standard of care

Note: All sensitivity analyses were performed with the PoC3 algorithm (PoC testing for initial test, PoC for confirmatory test, PoC test for tie-breaker test in the event of a discrepancy between the initial and confirmatory test) and primary implementation model. For the primary analysis all parameters were set to their baseline value (see Supplemental Table 1). Low and high PMTCT refers to the proportion of women receiving antiretroviral drugs to prevent mother-to-child transmission: low PMTCT=73%; high PMTCT=99% (compared to 93% in the primary analysis).
