# Supplementary material for: The cost-effectiveness of scaling-up rapid point-of-care testing for early infant diagnosis of HIV in southern Zambia
Source: PLoS One. 2021 Mar 9;16(3):e0248217. doi: 10.1371/journal.pone.0248217 (PMC7943017; doi:10.1371/journal.pone.0248217)
Supplement: S5 Table — (DOCX) [file pone.0248217.s007.docx]

**S5 Table. Sensitivity analysis of external factors influencing health outcomes and costs**

|  | **Primary analysis** | | | **Improved SoC** | | | **Worse SoC** | | |
| --- | --- | --- | --- | --- | --- | --- | --- | --- | --- |
|  | **SoC** | **GeneXpert** | **m-PIMA** | **SoC** | **GeneXpert** | **m-PIMA** | **SoC** | **GeneXpert** | **m-PIMA** |
| **HEALTH OUTCOMES** |  |  |  |  |  |  |  |  |  |
| **ART within 60 days** |  |  |  |  |  |  |  |  |  |
| Number | 470 | 1,377 | 1,400 | 674 | 1,377 | 1,400 | 204 | 1,377 | 1,400 |
| % | 27.8 | 81.4 | 82.8 | 39.8 | 81.4 | 82.8 | 12.0 | 81.4 | 82.8 |
| Additional compared to SoC | n/a | 907 | 930 | n/a | 703 | 726 | n/a | 1,173 | 1,197 |
| **Treated by 12 months** |  |  |  |  |  |  |  |  |  |
| Number | 862 | 1,438 | 1,463 | 1,019 | 1,438 | 1,463 | 549 | 1,438 | 1,463 |
| % | 50.9 | 85.0 | 86.4 | 60.2 | 85.0 | 86.4 | 32.4 | 85.0 | 86.4 |
| Additional compared to SoC | n/a | 576 | 601 | n/a | 420 | 444 | n/a | 890 | 914 |
| **Deaths** |  |  |  |  |  |  |  |  |  |
| Number | 307 | 71 | 65 | 254 | 71 | 65 | 376 | 71 | 65 |
| % | 18.1 | 4.2 | 3.8 | 15.0 | 4.2 | 3.8 | 22.2 | 4.2 | 3.8 |
| Averted compared to SoC | n/a | 236 | 242 | n/a | 183 | 189 | n/a | 305 | 311 |
| **False diagnoses** |  |  |  |  |  |  |  |  |  |
| % among children on ART | 0.00 | 0.01 | 0.00 | 0.00 | 0.01 | 0.00 | 0.00 | 0.01 | 0.00 |
| **COSTS** |  |  |  |  |  |  |  |  |  |
| Capital costs | $129,907 | $860,857 | $801,680 | $129,907 | $860,857 | $801,680 | $129,907 | $860,857 | $801,680 |
| Recurrent costs | $2,749,175 | $2,039,358 | $3,522,788 | $2,755,099 | $2,039,358 | $3,522,788 | $2,737,327 | $2,039,358 | $3,522,788 |
| Total program costs | $2,879,081 | $2,900,215 | $4,324,468 | $2,885,005 | $2,900,215 | $4,324,468 | $2,867,233 | $2,900,215 | $4,324,468 |
| **ICERs ($ per additional child)** |  |  |  |  |  |  |  |  |  |
| ART within 60 days | n/a | $23 | $1,554 | n/a | $22 | $1,982 | n/a | $28 | $1,218 |
| ART by 12 months | n/a | $37 | $2,406 | n/a | $36 | $3,243 | n/a | $37 | $1,594 |
| Deaths averted | n/a | $90 | $5,976 | n/a | $83 | $7,622 | n/a | $108 | $4,683 |

ART: antiretroviral therapy; ICER: incremental cost-effectiveness ratio; PoC: point-of-care testing; SoC: standard of care testing

Note: All sensitivity analyses were performed with the PoC3 algorithm (PoC testing for initial test, PoC for confirmatory test, PoC test for tie-breaker test in the event of a discrepancy between the initial and confirmatory test) and primary implementation model. For the primary analysis all parameters were set to their baseline value (see Supplemental Table 1). Improved and worse SoC refers to the proportion of children initiating ART within 60 days and by 12 months of age after SoC: Improved=43% and 65%; and worse=13% and 35% (compared to 30% and 55% in the primary analysis).

**S5 Table. Sensitivity analysis of external factors influencing health outcomes and costs, continued**

|  | **Primary analysis** | | | **Early testing cohort** | | | **Later testing cohort** | | |
| --- | --- | --- | --- | --- | --- | --- | --- | --- | --- |
|  | **SoC** | **GeneXpert** | **m-PIMA** | **SoC** | **GeneXpert** | **m-PIMA** | **SoC** | **GeneXpert** | **m-PIMA** |
| **HEALTH OUTCOMES** |  |  |  |  |  |  |  |  |  |
| **ART within 60 days** |  |  |  |  |  |  |  |  |  |
| Number | 470 | 1,377 | 1,400 | 394 | 1,158 | 1,175 | 678 | 1,889 | 1,928 |
| % | 27.8 | 81.4 | 82.8 | 26.3 | 77.2 | 78.4 | 29.2 | 84.7 | 86.4 |
| Additional compared to SoC | n/a | 907 | 930 | n/a | 763 | 781 | n/a | 1,211 | 1,250 |
| **Treated by 12 months** |  |  |  |  |  |  |  |  |  |
| Number | 862 | 1,438 | 1,463 | 723 | 1,209 | 1,227 | 1,243 | 1,973 | 2,013 |
| % | 50.9 | 85.0 | 86.4 | 48.2 | 80.6 | 81.9 | 53.4 | 88.5 | 90.3 |
| Additional compared to SoC | n/a | 576 | 601 | n/a | 486 | 505 | n/a | 730 | 770 |
| **Deaths** |  |  |  |  |  |  |  |  |  |
| Number | 307 | 71 | 65 | 277 | 72 | 67 | 365 | 74 | 66 |
| % | 18.1 | 4.2 | 3.8 | 18.5 | 4.8 | 4.5 | 15.7 | 3.3 | 2.9 |
| Averted compared to SoC | n/a | 236 | 242 | n/a | 205 | 210 | n/a | 290 | 299 |
| **False diagnoses** |  |  |  |  |  |  |  |  |  |
| % among children on ART | 0.00 | 0.01 | 0.00 | 0.00 | 0.01 | 0.00 | 0.00 | 0.00 | 0.00 |
| **COSTS** |  |  |  |  |  |  |  |  |  |
| Capital costs | $129,907 | $860,857 | $801,680 | $129,907 | $860,857 | $801,680 | $129,907 | $860,857 | $801,680 |
| Recurrent costs | $2,749,175 | $2,039,358 | $3,522,788 | $3,155,306 | $2,334,542 | $4,032,758 | $2,211,621 | $1,690,420 | $2,920,303 |
| Total program costs | $2,879,081 | $2,900,215 | $4,324,468 | $3,285,212 | $3,195,400 | $4,834,437 | $2,341,527 | $2,551,277 | $3721,983 |
| **ICERs ($ per additional child)** |  |  |  |  |  |  |  |  |  |
| ART within 60 days | n/a | $23 | $1,554 | n/a | -$118 | $1,984 | n/a | $173 | $1,105 |
| ART by 12 months | n/a | $37 | $2,406 | n/a | -$185 | $3,071 | n/a | $287 | $1,792 |
| Deaths averted | n/a | $90 | $5,976 | n/a | -$438 | $7,393 | n/a | $723 | $4,615 |

ART: antiretroviral therapy; ICER: incremental cost-effectiveness ratio; PoC: point-of-care testing; SoC: standard of care testing

Note: All sensitivity analyses were performed with the PoC3 algorithm (PoC testing for initial test, PoC for confirmatory test, PoC test for tie-breaker test in the event of a discrepancy between the initial and confirmatory test) and primary implementation model. For the primary analysis all parameters were set to their baseline value (see Supplemental Table 1). Early and late testing cohort refers to the distribution of infants entering the model for testing at different ages: early=80% entering at birth, 10% entering at 6 weeks, and 10% entering at 6 months of age; late=15% entering at birth, 45% entering at 6 weeks, and 40% entering at 6 months of age (compared to 40% entering at birth, 45% entering at 6 weeks, and 15% entering at 6 months of age in the primary analysis).

**S5 Table. Sensitivity analysis of external factors influencing health outcomes and costs, continued**

|  | **Primary analysis** | | | **Retention rate** | | | | | |
| --- | --- | --- | --- | --- | --- | --- | --- | --- | --- |
|  |  |  |  | **Low** | | | **High** | | |
|  | **SoC** | **GeneXpert** | **m-PIMA** | **SoC** | **GeneXpert** | **m-PIMA** | **SoC** | **GeneXpert** | **m-PIMA** |
| **HEALTH OUTCOMES** |  |  |  |  |  |  |  |  |  |
| **ART within 60 days** |  |  |  |  |  |  |  |  |  |
| Number | 470 | 1,377 | 1,400 | 460 | 1,345 | 1,369 | 481 | 1,411 | 1,433 |
| % | 27.8 | 81.4 | 82.8 | 27.2 | 79.5 | 80.9 | 28.4 | 83.4 | 84.7 |
| Additional compared to SoC | n/a | 907 | 930 | n/a | 885 | 909 | n/a | 930 | 952 |
| **Treated by 12 months** |  |  |  |  |  |  |  |  |  |
| Number | 862 | 1,438 | 1,463 | 843 | 1,405 | 1,430 | 882 | 1,474 | 1,497 |
| % | 50.9 | 85.0 | 86.4 | 49.8 | 83.0 | 84.5 | 52.1 | 87.1 | 88.4 |
| Additional compared to SoC | n/a | 576 | 601 | n/a | 561 | 587 | n/a | 592 | 615 |
| **Deaths** |  |  |  |  |  |  |  |  |  |
| Number | 307 | 71 | 65 | 307 | 76 | 70 | 306 | 66 | 59 |
| % | 18.1 | 4.2 | 3.8 | 18.1 | 4.5 | 4.1 | 18.1 | 3.9 | 3.5 |
| Averted compared to SoC | n/a | 236 | 242 | n/a | 231 | 237 | n/a | 241 | 247 |
| **False diagnoses** |  |  |  |  |  |  |  |  |  |
| % among children on ART | 0.00 | 0.01 | 0.00 | 0.00 | 0.01 | 0.00 | 0.00 | 0.01 | 0.00 |
| **COSTS** |  |  |  |  |  |  |  |  |  |
| Capital costs | $129,907 | $860,857 | $801,680 | $129,907 | $860,857 | $801,680 | $129,907 | $860,857 | $801,680 |
| Recurrent costs | $2,749,175 | $2,039,358 | $3,522,788 | $2,632,102 | $1,952,782 | $3,373,403 | $2,872,038 | $2,130,210 | $3,679,556 |
| Total program costs | $2,879,081 | $2,900,215 | $4,324,468 | $2,762,008 | $2,813,639 | $4,175,083 | $3,001,945 | $2,991,068 | $4,481,236 |
| **ICERs ($ per additional child)** |  |  |  |  |  |  |  |  |  |
| ART within 60 days | n/a | $23 | $1,554 | n/a | $58 | $1,554 | n/a | -$12 | $1,554 |
| ART by 12 months | n/a | $37 | $2,406 | n/a | $92 | $2,408 | n/a | -$18 | $2,405 |
| Deaths averted | n/a | $90 | $5,976 | n/a | $224 | $5,961 | n/a | -$45 | $5,993 |

ART: antiretroviral therapy; ICER: incremental cost-effectiveness ratio; PoC: point-of-care testing; SoC: standard of care testing

Note: All sensitivity analyses were performed with the PoC3 algorithm (PoC testing for initial test, PoC for confirmatory test, PoC test for tie-breaker test in the event of a discrepancy between the initial and confirmatory test) and primary implementation model. For the primary analysis all parameters were set to their baseline value (see Supplemental Table 1). Retention rate refers to the proportion of children returning for subsequent testing at a later age: low/high=75%/85% for mothers who received PMTCT and 35%/85% for mothers who did not receive PMTCT (compared to 80% for mothers who received PMTCT and 60% for mothers who did not receive PMTCT in the primary analysis).
