# Supplementary material for: The cost-effectiveness of scaling-up rapid point-of-care testing for early infant diagnosis of HIV in southern Zambia
Source: PLoS One. 2021 Mar 9;16(3):e0248217. doi: 10.1371/journal.pone.0248217 (PMC7943017; doi:10.1371/journal.pone.0248217)
Supplement: S6 Table — (DOCX) [file pone.0248217.s008.docx]

**S6 Table. Health outcomes and costs by implementation model**

|  | **SoC** | **GeneXpert** | | | **m-PIMA** | |
| --- | --- | --- | --- | --- | --- | --- |
|  |  | **Primary** | **Expanded** | **Hub-and-spoke** | **Primary** | **Expanded** |
| **MODEL OUTPUTS** |  |  |  |  |  |  |
| Number of HIV-infected children | 1692 | 1,692 | 1,692 | 1,692 | 1,692 | 1,692 |
| Number of HIV-infected children diagnosed | 1,567 | 1,530 | 1,538 | 1,530 | 1,556 | 1,558 |
| Number of SoC tests | 72,207 | 0 | 16,608 | 0 | 0 | 16,608 |
| Number of PoC tests | 0 | 73,065 | 56,260 | 73,065 | 72,963 | 56,181 |
| **HEALTH OUTCOMES** |  |  |  |  |  |  |
| **ART within 60 days** |  |  |  |  |  |  |
| Number | 470 | 1,377 | 1,169 | 1,285 | 1,400 | 1,186 |
| % | 27.8 | 81.4 | 69.1 | 76.0 | 82.8 | 70.1 |
| Additional compared to SoC | n/a | 907 | 698 | 815 | 930 | 716 |
| **Treated by 12 months** |  |  |  |  |  |  |
| Number | 862 | 1,438 | 1,306 | 1,347 | 1,463 | 1,324 |
| % | 50.9 | 85.0 | 77.2 | 79.6 | 86.4 | 78.3 |
| Additional compared to SoC | n/a | 576 | 444 | 485 | 601 | 462 |
| **Deaths** |  |  |  |  |  |  |
| Number | 307 | 71 | 125 | 95 | 65 | 120 |
| % | 18.1 | 4.2 | 7.4 | 5.6 | 3.8 | 7.1 |
| Averted compared to SoC | n/a | 236 | 181 | 212 | 242 | 186 |
| **False diagnoses** |  |  |  |  |  |  |
| % among children on ART | 0.00 | 0.01 | 0.01 | 0.01 | 0.00 | 0.00 |
| **COSTS** |  |  |  |  |  |  |
| Capital costs | $129,907 | $860,857 | $1,735,337 | $866,233 | $801,680 | $1,613,015 |
| Recurrent costs | $2,749,175 | $2,039,358 | $2,202,616 | $2,130,785 | $3,522,788 | $3,344,857 |
| Total program costs | $2,879,081 | $2,900,215 | $3,937,953 | $2,997,018 | $4,324,468 | $4,957,872 |
| **ICERs ($ per additional child)** |  |  |  |  |  |  |
| ART within 60 days | n/a | $23 | $1,516 | $145 | $1,554 | $2,902 |
| ART by 12 months | n/a | $37 | $2,386 | $243 | $2,406 | $4,495 |
| Deaths averted | n/a | $90 | $5,838 | $557 | $5,976 | $11,163 |

ART: antiretroviral therapy; ICER: incremental cost effectiveness ratio; n/a: not applicable; PoC: point-of-care; SoC: standard of care

Note: Results are reported for the PoC3 testing algorithm (PoC testing for initial test, PoC for confirmatory test, PoC test for tie-breaker test in the event of a discrepancy between the initial and confirmatory test), as this algorithm was the dominant in the analysis comparing testing algorithms. The primary implementation model included placement of PoC platforms at 40 facilities assumed to cover approximately 60% of the HIV-exposed population (see Supplementary Materials for selection rationale). All other HIV-exposed infants requiring EID were assumed to be referred to these facilities for testing. The second implementation model included expanded access to PoC testing with PoC platforms placed at 74 facilities assumed to cover 77% of the HIV-exposed infant population, with the remaining 23% tested under the SoC. The third implementation model was a hub-and-spoke approach, with the 40 facilities from the primary model serving as PoC testing hubs for the 60% of HIV-exposed infants served by the hubs. For the remaining 40% of the population, dried blood spot cards were transported to the hubs for testing. As the m-PIMA is not currently approved for use with dried blood spot cards, this model was only considered for GeneXpert.
