# Supplementary material for: The cost-effectiveness of scaling-up rapid point-of-care testing for early infant diagnosis of HIV in southern Zambia
Source: PLoS One. 2021 Mar 9;16(3):e0248217. doi: 10.1371/journal.pone.0248217 (PMC7943017; doi:10.1371/journal.pone.0248217)
Supplement: S7 Table. Multivariable sensitivity analysis: A) parameter values and B) health outcomes and costs — (DOCX) [file pone.0248217.s009.docx]

**S7A Table. Multivariable sensitivity analysis – parameter values**

|  | **Primary analysis value** | **Primary PMTCT coverage** | | **Low PMTCT coverage** | | **High PMTCT coverage** | |
| --- | --- | --- | --- | --- | --- | --- | --- |
|  |  | **Worst case value** | **Best case value** | **Worst case value** | **Best case value** | **Worst case value** | **Best case value** |
| **PARAMETER VALUES** |  |  |  |  |  |  |  |
| **PMTCT coverage** | 0.93 | 0.93 | 0.93 | 0.73 | 0.73 | 0.99 | 0.99 |
| **Probability of ART initiation** |  |  |  |  |  |  |  |
| After SoC testing, within 60 days | 0.30 | 0.43 | 0.13 | 0.43 | 0.13 | 0.43 | 0.13 |
| After SoC testing, by 12 months of age | 0.55 | 0.65 | 0.35 | 0.65 | 0.35 | 0.65 | 0.35 |
| **Probability of returning for subsequent EID testing** |  |  |  |  |  |  |  |
| Mother received PMTCT | 0.80 | 0.75 | 0.85 | 0.75 | 0.85 | 0.75 | 0.85 |
| Mother did not receive PMTCT | 0.60 | 0.35 | 0.85 | 0.35 | 0.85 | 0.35 | 0.85 |
| **GeneXpert characteristics and costs** |  |  |  |  |  |  |  |
| Lifespan of instrument | 7 | 3 | 7 | 3 | 7 | 3 | 7 |
| Utilization rate | 1 | 1 | 0.1 | 1 | 0.1 | 1 | 0.1 |
| Time spent to run each test | 0.6 | 0.5 | 0.17 | 0.5 | 0.17 | 0.5 | 0.17 |
| **m-PIMA characteristics and costs** |  |  |  |  |  |  |  |
| Lifespan | 5 | 2 | 5 | 2 | 5 | 2 | 5 |
| Utilization rate | 1 | 1 | 0.15 | 1 | 0.15 | 1 | 0.15 |
| Time spent to run each test | 0.6 | 0.5 | 0.17 | 0.5 | 0.17 | 0.5 | 0.17 |
| **Cost of staff time for blood collection** |  |  |  |  |  |  |  |
| Time spent per test on sample collection (includes pre-test counseling) | 0.6 | 1 | 0.5 | 1 | 0.5 | 1 | 0.5 |
| **PoC sensitivity** |  |  |  |  |  |  |  |
| GeneXpert | 0.968 | 0.9268 | 0.9895 | 0.9268 | 0.9895 | 0.9268 | 0.9895 |
| m-PIMA | 0.99 | 0.9645 | 0.9988 | 0.9645 | 0.9988 | 0.9645 | 0.9988 |
| **Proportion of children entering the cohort** |  |  |  |  |  |  |  |
| At birth | 0.4 | 0.4 | 0.4 | 0.15 | 0.15 | 0.8 | 0.8 |
| At 6 weeks of age | 0.45 | 0.45 | 0.45 | 0.45 | 0.45 | 0.1 | 0.1 |
| At 6 months of age | 0.15 | 0.15 | 0.15 | 0.4 | 0.4 | 0.1 | 0.1 |
| **SoC characteristics - lifespan** | 5 | 7 | 5 | 7 | 5 | 7 | 5 |

ART: antiretroviral therapy; PMTCT: prevention of mother-to-child transmission; SoC: standard of care

**S7B Table. Multivariable sensitivity analysis - health outcomes and costs**

|  | **Primary analysis** | | | **Multivariate sensitivity analysis: Primary PMTCT coverage** | | | | | |
| --- | --- | --- | --- | --- | --- | --- | --- | --- | --- |
|  |  |  |  | **Worst case** | | | **Best case** | | |
|  | **SoC** | **GeneXpert** | **m-PIMA** | **SoC** | **GeneXpert** | **m-PIMA** | **SoC** | **GeneXpert** | **m-PIMA** |
| **HEALTH OUTCOMES** |  |  |  |  |  |  |  |  |  |
| **ART within 60 days** |  |  |  |  |  |  |  |  |  |
| Number | 470 | 1,377 | 1,400 | 659 | 1296 | 1341 | 208 | 1432 | 1441 |
| % | 27.8 | 81.4 | 82.8 | 39.0 | 76.6 | 79.2 | 12.3 | 84.7 | 85.2 |
| Additional compared to SoC | n/a | 907 | 930 | n/a | 637 | 682 | n/a | 1224 | 1233 |
| **Treated by 12 months** |  |  |  |  |  |  |  |  |  |
| Number | 862 | 1,438 | 1,463 | 997 | 1354 | 1400 | 561 | 1496 | 1505 |
| % | 50.9 | 85.0 | 86.4 | 58.9 | 80.0 | 82.8 | 33.2 | 88.4 | 89.0 |
| Additional compared to SoC | n/a | 576 | 601 | n/a | 357 | 404 | n/a | 935 | 944 |
| **Deaths** |  |  |  |  |  |  |  |  |  |
| Number | 307 | 71 | 65 | 255 | 89 | 77 | 377 | 60 | 57 |
| % | 18.1 | 4.2 | 3.8 | 15.1 | 5.3 | 4.6 | 22.3 | 3.5 | 3.4 |
| Averted compared to SoC | n/a | 236 | 242 | n/a | 166 | 178 | n/a | 317 | 320 |
| **False diagnoses** |  |  |  |  |  |  |  |  |  |
| % among children on ART | 0.00 | 0.01 | 0.00 | 0.00 | 0.10 | 0.01 | 0.00 | 0.11 | 0.01 |
| **COSTS** |  |  |  |  |  |  |  |  |  |
| Capital costs | $129,907 | $860,857 | $801,680 | $98,430 | $2,008,667 | $2,004,200 | $129,907 | $86,086 | $120,252 |
| Recurrent costs | $2,749,175 | $2,039,358 | $3,522,788 | $2,774,644 | $2,388,463 | $3,616,198 | $2,822,857 | $1,989,208 | $3,606,036 |
| Total program costs | $2,879,081 | $2,900,215 | $4,324,468 | $2,873,074 | $4,397,130 | $5,621,398 | $2,952,764 | $2,075,294 | $3,726,288 |
| **ICERs ($ per additional child)** |  |  |  |  |  |  |  |  |  |
| ART within 60 days | n/a | $23 | $1,554 | n/a | $2,394 | $4,031 | n/a | -$717 | $627 |
| ART by 12 months | n/a | $37 | $2,406 | n/a | $4,269 | $6,803 | n/a | -$938 | $819 |
| Deaths averted | n/a | $90 | $5,976 | n/a | $9,201 | $15,475 | n/a | -$2765 | $2,418 |

ART: antiretroviral therapy; ICER: incremental cost effectiveness ratio; n/a: not applicable; PMTCT: prevention of mother-to-child transmission; PoC: point-of-care; SoC: standard of care

Note: Results are reported for the PoC3 testing algorithm (PoC testing for initial test, PoC for confirmatory test, PoC test for tie-breaker test in the event of a discrepancy between the initial and confirmatory test), as this algorithm was the dominant in the analysis comparing testing algorithms. The primary implementation model included placement of PoC platforms at 40 facilities assumed to cover approximately 60% of the HIV-exposed population (see Supplementary Materials for selection rationale). All other HIV-exposed infants requiring EID were assumed to be referred to these facilities for testing. Primary PMTCT coverage = 93%.

**S7B Table. Multivariable sensitivity analysis - health outcomes and costs, continued**

|  | **Univariate sensitivity analysis:**  **High PMTCT coverage** | | | **Multivariate sensitivity analysis: High PMTCT coverage** | | | | | |
| --- | --- | --- | --- | --- | --- | --- | --- | --- | --- |
|  |  |  |  | **Worst case** | | | **Best case** | | |
|  | **SoC** | **GeneXpert** | **m-PIMA** | **SoC** | **GeneXpert** | **m-PIMA** | **SoC** | **GeneXpert** | **m-PIMA** |
| **HEALTH OUTCOMES** |  |  |  |  |  |  |  |  |  |
| **ART within 60 days** |  |  |  |  |  |  |  |  |  |
| Number | 385 | 1,127 | 1,146 | 484 | 959 | 988 | 157 | 1082 | 1088 |
| % | 27.5 | 80.6 | 82.0 | 36.1 | 71.4 | 73.6 | 11.7 | 80.6 | 81.1 |
| Additional compared to SoC | n/a | 742 | 761 | n/a | 474 | 504 | n/a | 925 | 931 |
| **Treated by 12 months** |  |  |  |  |  |  |  |  |  |
| Number | 705 | 1,177 | 1,197 | 732 | 1001 | 1032 | 424 | 1130 | 1137 |
| % | 50.4 | 84.2 | 85.6 | 54.5 | 74.6 | 76.9 | 31.6 | 84.2 | 84.7 |
| Additional compared to SoC | n/a | 472 | 492 | n/a | 269 | 300 | n/a | 706 | 713 |
| **Deaths** |  |  |  |  |  |  |  |  |  |
| Number | 246 | 59 | 54 | 205 | 79 | 71 | 299 | 55 | 54 |
| % | 17.5 | 4.2 | 3.9 | 15.3 | 5.9 | 5.3 | 22.3 | 4.1 | 4.0 |
| Averted compared to SoC | n/a | 187 | 192 | n/a | 126 | 134 | n/a | 244 | 245 |
| **False diagnoses** |  |  |  |  |  |  |  |  |  |
| % among children on ART | 0.00 | 0.02 | 0.00 | 0.00 | 0.12 | 0.01 | 0.00 | 0.13 | 0.01 |
| **COSTS** |  |  |  |  |  |  |  |  |  |
| Capital costs | $129,907 | $860,857 | $801,680 | $98,430 | $2,008,667 | $2,004,200 | $129,907 | $86,086 | $120,252 |
| Recurrent costs | $2,768,113 | $2,049,691 | $3,540,799 | $3,183,937 | $2,733,251 | $4,138,237 | $3,287,153 | $2,305,413 | $4,179,355 |
| Total program costs | $2,898,019 | $2,910,548 | $4,342,479 | $3,282,367 | $4,741,918 | $6,142,437 | $3,417,060 | $2,391,498 | $4,299,607 |
| **ICERs ($ per additional child)** |  |  |  |  |  |  |  |  |  |
| ART within 60 days | n/a | $17 | $1,897 | n/a | $3,076 | $5,677 | n/a | -$1,109 | $948 |
| ART by 12 months | n/a | $27 | $2,939 | n/a | $5,417 | $9,534 | n/a | -$1,452 | $1,238 |
| Deaths averted | n/a | $67 | $7,514 | n/a | $11,539 | $21,360 | n/a | -$4,211 | $3,600 |

ART: antiretroviral therapy; ICER: incremental cost effectiveness ratio; n/a: not applicable; PMTCT: prevention of mother-to-child transmission; PoC: point-of-care; SoC: standard of care

Note: Results are reported for the PoC3 testing algorithm (PoC testing for initial test, PoC for confirmatory test, PoC test for tie-breaker test in the event of a discrepancy between the initial and confirmatory test), as this algorithm was the dominant in the analysis comparing testing algorithms. The primary implementation model included placement of PoC platforms at 40 facilities assumed to cover approximately 60% of the HIV-exposed population (see Supplementary Materials for selection rationale). All other HIV-exposed infants requiring EID were assumed to be referred to these facilities for testing. Low PMTCT coverage = 73%.

**S7B Table. Multivariable sensitivity analysis - health outcomes and costs, continued**

|  | **Univariate sensitivity analysis:**  **Low PMTCT coverage** | | | **Multivariate sensitivity analysis: Low PMTCT coverage** | | | | | |
| --- | --- | --- | --- | --- | --- | --- | --- | --- | --- |
|  |  |  |  | **Worst case** | | | **Best case** | | |
|  | **SoC** | **GeneXpert** | **m-PIMA** | **SoC** | **GeneXpert** | **m-PIMA** | **SoC** | **GeneXpert** | **m-PIMA** |
| **HEALTH OUTCOMES** |  |  |  |  |  |  |  |  |  |
| **ART within 60 days** |  |  |  |  |  |  |  |  |  |
| Number | 743 | 2,178 | 2,214 | 1403 | 2725 | 2837 | 433 | 2974 | 2995 |
| % | 28.2 | 82.7 | 84.1 | 41.4 | 80.5 | 83.8 | 12.8 | 87.9 | 88.5 |
| Additional compared to SoC | n/a | 1,435 | 1,471 | n/a | 1323 | 1435 | n/a | 2541 | 2562 |
| **Treated by 12 months** |  |  |  |  |  |  |  |  |  |
| Number | 1,363 | 2,275 | 2,312 | 2120 | 2847 | 2963 | 1166 | 3106 | 3128 |
| % | 51.8 | 86.4 | 87.8 | 62.7 | 84.1 | 87.6 | 34.5 | 91.8 | 92.4 |
| Additional compared to SoC | n/a | 912 | 950 | n/a | 726 | 843 | n/a | 1940 | 1963 |
| **Deaths** |  |  |  |  |  |  |  |  |  |
| Number | 499 | 109 | 98 | 466 | 150 | 123 | 702 | 95 | 90 |
| % | 19.0 | 4.1 | 3.7 | 13.8 | 4.4 | 3.6 | 20.7 | 2.8 | 2.6 |
| Averted compared to SoC | n/a | 390 | 401 | n/a | 316 | 343 | n/a | 606 | 612 |
| **False diagnoses** |  |  |  |  |  |  |  |  |  |
| % among children on ART | 0.00 | 0.01 | 0.00 | 0.00 | 0.08 | 0.01 | 0.00 | 0.08 | 0.01 |
| **COSTS** |  |  |  |  |  |  |  |  |  |
| Capital costs | $129,907 | $860,857 | $801,680 | $98,430 | $2,008,667 | $2,004,200 | $129,907 | $86,086 | $120,252 |
| Recurrent costs | $2,687,677 | $2,005,804 | $3,464,299 | $2,269,749 | $1,976,986 | $2,993,794 | $2,280,688 | $1,641,179 | $2,975,055 |
| Total program costs | $2,817,583 | $2,866,661 | $4,265,979 | $2,368,179 | $3,985,653 | $4,997,994 | $2,410,595 | $1,727,265 | $3,095,307 |
| **ICERs ($ per additional child)** |  |  |  |  |  |  |  |  |  |
| ART within 60 days | n/a | $34 | $985 | n/a | $1,223 | $1,833 | n/a | -$269 | $267 |
| ART by 12 months | n/a | $54 | $1,525 | n/a | $2,227 | $3,120 | n/a | -$352 | $349 |
| Deaths averted | n/a | $126 | $5,976 | n/a | $5,120 | $7,668 | n/a | -$1,269 | $1,119 |

ART: antiretroviral therapy; ICER: incremental cost effectiveness ratio; n/a: not applicable; PMTCT: prevention of mother-to-child transmission; PoC: point-of-care; SoC: standard of care

Note: Results are reported for the PoC3 testing algorithm (PoC testing for initial test, PoC for confirmatory test, PoC test for tie-breaker test in the event of a discrepancy between the initial and confirmatory test), as this algorithm was the dominant in the analysis comparing testing algorithms. The primary implementation model included placement of PoC platforms at 40 facilities assumed to cover approximately 60% of the HIV-exposed population (see Supplementary Materials for selection rationale). All other HIV-exposed infants requiring EID were assumed to be referred to these facilities for testing. High PMTCT coverage = 99%.
