# Supplementary material for: The cost-effectiveness of scaling-up rapid point-of-care testing for early infant diagnosis of HIV in southern Zambia
Source: PLoS One. 2021 Mar 9;16(3):e0248217. doi: 10.1371/journal.pone.0248217 (PMC7943017; doi:10.1371/journal.pone.0248217)
Supplement: Attachment — Submitted filename: Response to reviewers_05feb2021.pdf [file pone.0248217.s010.pdf]

Reviewer #1:

4. The authors have not defined the time and period of study.

The model was run over a five-year time horizon (as outlined on lines 106-107) and is anchored in 2018 when the majority of the costs were collected (as outlined on lines 179-181 and 192).

Data were collected in two studies (NSEBA study and EID study) with details provided primarily in the Supplementary Methods. To assist the reader, the period of performance for each study was added to the main text of the manuscript (lines 167-169).

5. I could not find reference no 38 in the manuscript.

We thank the reviewer for identifying this omission. We have corrected the issue and reference 38 is now included on line 194.

6. The discussion could include a line or two about incidence of Tuberculosis in Southern Province, Zambia to account for use of PoC platform geneXpert across diseases.

The discussion was revised to include a sentence about the utility of multi-disease platforms in high burden setting, with a reference to the burden of tuberculosis and HIV in Zambia (lines 369-372).

7. Line 149 to 152 - The selection criteria should be included in the body of the manuscript rather than in the supplementary data.

The selection criteria for the primary and expanded access implementation models were added to the main text of the manuscript (lines 149-150 and 154-155).

Reviewer #2:

The design and outcomes of this study is adequate to meet the stated objectives, congratulations to the authors, however, they should consider including in the abstract and discussion sections the fact that point of care testing should be considered and seen to occur within an integrated diagnostic optimized network and complimentarily with SoC in infants diagnosis. It is not in every setting that point of care platforms must be used for infant diagnose; the decision to use point of care should be informed by the outcomes of an optimized network, addressing all systemic issues and prioritizing public health impact of quick diagnosis and access to care of these infants. In effect, where SoC is the most appropriate platform for diagnosis, for example when large sample volumes are to be tested, systemic challenges to include long turnaround time for the return of results should be addressed rather that substituting for point of care. This study itself confirms the fact that SoC is not more expensive when compared with point of care.

We agree with the Reviewer and thank them for raising this point. PoC testing is not feasible or desirable in all settings, and where implemented it will need to be incorporated into an overall program that still includes SoC testing. The cost-effectiveness of PoC testing depends on many factors, including how well the SoC performs. This was demonstrated in our sensitivity analysis, particularly for the m-PIMA platform. SoC testing may be the best option for certain settings or may continue to be part of the testing algorithm where PoC testing is implemented. Either way, implementation of PoC testing should not be viewed as a decision to stop working to improve

laboratory testing. A functional and efficient laboratory system will have benefits for EID as well as many other conditions. The Discussion section was revised to include these points (lines 389 to 397).
